# Zinc Alleviates Gut Barrier Dysfunction by Promoting the Methylation of AKT

**DOI:** 10.1002/advs.202508280

**Published:** 2025-07-11

**Authors:** Chuanjiang Cai, Yining Zheng, Bo Sun, Guoyan Wang, Pengfei Li, Huijun Geng, Rongnuo Li, Miaomiao Zhu, Yuanyuan Zhu, Dingping Feng, Lei Chen, Guiyan Chu, Lu Deng, Shiyan Qiao

**Affiliations:** ^1^ College of Animal Science and Technology Northwest A&F University Yangling Shaanxi 712100 China; ^2^ Shenzhen Research Institute Northwest A&F University Shenzhen Guangdong 518000 China; ^3^ State Key Laboratory of Animal Nutrition and Feeding College of Animal Science and Technology China Agricultural University Beijing 100193 China

**Keywords:** AKT methylation, gut barrier, METAP1, zinc

## Abstract

Zinc plays a crucial role in the gut barrier function and are widely used for the prevention of bowel disease. However, the mechanism via which zinc supplementation exerts this regulatory effect is unclear. The present study identifies and characterizes the zinc‐responsive activation of AKT and demonstrates its function in alleviating gut barrier dysfunction. Mechanistically, zinc increased intracellular SAM production, a methyl donor, by promoting the activation of the metallochaperone ZNG1‐METAP1 complex. Subsequently, zinc facilitates methylation (symmetrical dimethylarginine, SDMA) of AKT at residues R391 and R15, which is facilitated by PRMT5. The AKT^SDMA^ modification promotes AKT translocation from the cytoplasm to the plasma membrane and its interaction with mTORC2, ultimately promoting AKT activation and cell proliferation. Notably, histidine has an antagonistic effect on zinc‐induced the AKT activation, cell proliferation, and gut barrier improvement by chelating zinc. These results demonstrate that zinc activates AKT and alleviates gut barrier dysfunction by inducing activation of the ZNG1‐METAP1‐PRMT5‐AKT^SDMA^ pathway, and highlight that limiting histidine intake may have effective therapeutic potential for bowel diseases such as Crohn's disease and Ulcerative colitis.

## Graphical abstract

Zinc activates the ZNG1‐METAP1 complex to elevate intracellular SAM levels, which promotes PRMT5‐mediated symmetrical dimethylation of AKT at R391 and R15. This epigenetic modification facilitates AKT membrane translocation and mTORC2 activation, thereby enhancing epithelial proliferation and gut barrier integrity. Notably, histidine antagonizes this zinc‐driven pathway by chelating zinc.

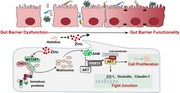

## 1 Introduction

The gut barrier serves as the primary defense mechanism separating the gut from the external environment. It plays an important role in the selective entry of nutrients into the human body, separates the intestinal epithelium from the luminal contents and prevents the entry of bacteria, antigens and other harmful substances into the luminal lamina propria of the intestinal mucosa.^[^ ^1^ ^]^ The tight junction, composed of ZO‐1, occludin, cadherin, and claudin, etc., is an important structure of the gut barrier,^[^ ^1^, ^2^ ^]^ and its dysfunction leads to intestinal leakage and inflammation, which is an important factor in the development of intestinal diseases such as Crohn's disease and Ulcerative colitis.^[^ ^3^ ^]^ However, the aetiology of impaired gut barrier or tight junction of the gut and its triggering bowel disease mechanisms remain enigmatic,^[^ ^4^ ^]^ and the search for interventions that alleviate gut barrier dysfunction may provide better options for the treatment of bowel diseases such as Crohn's disease and Ulcerative colitis.

Zinc is essential for the proper development and differentiation of intestinal epithelial cells and for maintaining the integrity of the gut barrier.^[^ ^5^ ^]^ However, the importance of zinc has not yet been fully recognized, and ≈50% of the global population is still at risk of inadequate zinc intake.^[^ ^6^ ^]^ Zinc deficiency attenuates intestinal stem cell growth, impairs intestinal tight junctions and results in intestinal epithelial leakage.^[^ ^7^ ^]^ This may be the reason why zinc supplementation has been widely used for the improvement of gut barrier function and the prevention of diarrhea in children and farm animals.^[^ ^5^, ^8^ ^]^ The intracellular zinc concentration is controlled by regulation of the zinc‐specific transporters (e.g., the ZIP and ZNT family), zinc sensors (e.g., GPR39), and zinc‐binding proteins (e.g., metallothioneins).^[^ ^9^ ^]^ Recently, a conserved zinc metallochaperone protein, namely, ZNG1, has been identified, which can directly transfer zinc to the methionine aminopeptidase METAP1, thus activating METAP1.^[^ ^10^ ^]^ METAP1 is mainly responsible for cleaving the first amino acid (usually methionine) in immature proteins, which is crucial for protein modification and maturation.^[^ ^11^ ^]^ In addition, previous studies have reported that zinc can activate AKT signaling in various contexts, which plays a critical role in regulating barrier‐associated protein expression (e.g., ZO‐1, occludin, claudin‐1, and MUC2) and intestinal epithelial cell proliferation, and is essential for maintaining gut function.^[^ ^12^ ^]^ For instance, it has been shown to promote preadipocyte proliferation and differentiation via the PI3K/AKT pathway, enhance cancer cell growth through redox‐regulated AKT activation, and act as a second messenger that induces rapid phosphorylation of AKT and ERK in response to zinc influx.^[^ ^13^ ^]^ Notably, recent research proposed an alternative zinc‐responsive pathway involving AHR (aryl hydrocarbon receptor), highlighting zinc's ability to improve gut function via AHR activation and stem cell maintenance.^[^ ^1a^ ^]^ While these findings supported zinc's beneficial role and initially established a link between zinc and AKT signaling, their focus remains on zinc uptake and immune cell regulation, the underlying molecular mechanism by which zinc activates AKT remained undefined. The AKT is recognized as a critical regulator of the signaling pathway composed of PI3K (phosphatidylinositol 3‐kinase) and PTEN (phosphatase and tensin homolog deleted on chromosome 10), which is involved in a signaling cascade that is frequently dysregulated in several diseases, including gut barrier dysfunction and diarrhea.^[^ ^14^ ^]^ Growth factors promote the activation of PI3K, which converts PIP2 to PIP3, thereby recruiting PDK1 (3‐phosphoinositide‐dependent protein kinase‐1), mTORC2 (mechanistic target of rapamycin complex 2), and AKT to the cell membrane. Subsequently, PDK1 and mTORC2 phosphorylate AKT at the Thr308 and Ser473 sites, respectively, and thereby, promote the activation of AKT.^[^ ^15^ ^]^ A recent study suggests that amino acid‐induced activation of the p38 pathway is also critical for AKT phosphorylation.^[^ ^16^ ^]^ Besides phosphorylation, methylation has been shown to play an important role in AKT activation. Studies have shown that methylation of AKT by the methyltransferase SETDB1 is critical for cell membrane localization, phosphorylation, and activation of AKT.^[^ ^17^ ^]^ Additionally, two recent reports pointed out that PRMT5 can specifically mediate AKT methylation at the R15K and R391 sites, thus promoting its localization to the cell membrane and subsequently promoting the activation of AKT.^[^ ^18^ ^]^

In this study, our data showed that zinc exerts its alleviating effects on gut inflammation and barrier dysfunction through the activation of AKT. We indicated that zinc supplementation facilitates the transfer of zinc to METAP1 via ZNG1 and promoting the production of S‐adenosyl methionine (SAM), which serves as a methyl donor and promotes PRMT5‐mediated AKT methylation that ultimately enhances AKT localization to the cell membrane and activation. In addition, we found that the histidine inhibits zinc‐mediated AKT activation and alleviation gut inflammation and barrier dysfunction by chelating zinc. Overall, our research reveals that the zinc‐AKT signaling axis holds significant regulatory implications in the context of gut barrier dysfunction.

## 2 Results

### 2.1 Role of Zinc in the Alleviation of Gut Barrier Dysfunction and Activation of AKT

**Figure 1:**
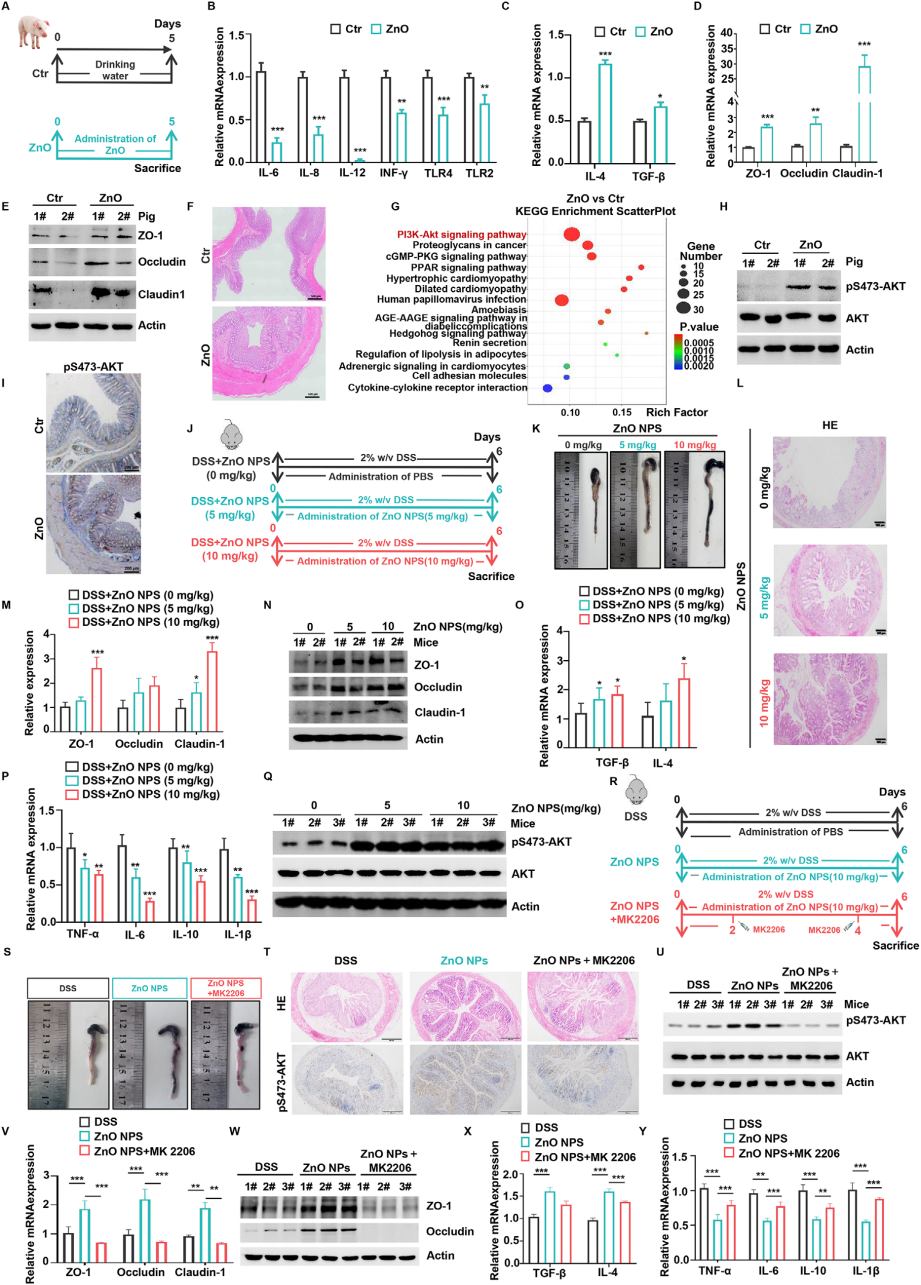
Role of zinc in the alleviation of gut barrier dysfunction and activation of AKT. A) Experimental flowchart for piglet. Piglets in the ZnO treatment group (n = 8) received a 5‐day ZnO supplementation diet, while the control group (n = 8) maintained normal feeding conditions. B) The expression levels of IL‐6, IL‐8, IL‐12, INF‐γ, TLR2, and TLR4 in the colon tissue of piglets from the control group and ZnO treatment group were detected by qRT‐PCR. C) The expression levels of IL‐4 and TGF‐β in the colon tissue of piglets from the control group and ZnO treatment group were detected by qRT‐PCR. D,E) The expression levels of ZO‐1, occludin and claudin‐1 in the colon tissue of piglets from the control group and ZnO treatment group were detected by qRT‐PCR (D) or WB (E). F) H&E staining of colon tissue in piglets from the control group and ZnO treatment group. G) KEGG enrichment analysis in colon tissue of piglets from the control group and ZnO treatment group. H,I) AKT activity in piglet colon tissue was detected by immunohistochemical staining (H) or WB (I). J) Flowchart of Experiment on mice. During the experiment, all mice were provided with drinking water treated with 2% DSS. The control group (n = 6) was gavaged with 0.2 ml of PBS. The DSS + ZnO NPs (5 mg kg^−1^) group and the DSS + ZnO NPs (10 mg kg^−1^) group were gavaged with the same amount of nano‐zinc oxide of different concentrations every day for 6 days. K) Colon length of the control group, the DSS + ZnO NPs (5 mg kg^−1^) group and the DSS + ZnO NPs (10 mg kg^−1^) group. L) H&E staining of colon tissue in mice from the control group, the DSS + ZnO NPs (5 mg kg^−1^) group and the DSS + ZnO NPs (10 mg kg^−1^) group. M,N) The expression levels of ZO‐1, occludin and claudin‐1 in the colon tissue of mice were detected by qRT‐PCR (M) or WB (N). O) The expression levels of TGF‐β and IL‐4 in the colon tissue of mice were detected by qRT‐PCR. P) The expression levels of TNF‐α, IL‐6, IL‐10, and IL‐1β in the colon tissue of mice were detected by qRT‐PCR. Q) AKT activity in mice colon tissue was detected by WB. R) Experimental design for mouse treatment. All mice received drinking water containing 2% DSS throughout the experiment. The control group (n = 6) was gavaged daily with 0.2 mL PBS. The DSS + ZnO NPs group received daily oral gavage of ZnO NPs (10 mg kg^−1^) for 6 days. The ZnO NPs + MK‐2206 group additionally received intraperitoneal injections of MK‐2206 (120 mg kg^−1^) on days 2 and 4. S) Colon length measurements in the DSS group, DSS + ZnO NPs group, and ZnO NPs + MK‐2206 group. T) Representative images of H&E staining and pS473‐AKT immunohistochemistry in colon tissues from each group. U) Western blot analysis of AKT activity in mouse colon tissue. V,W) Expression levels of tight junction proteins ZO‐1, Occludin, and Claudin‐1 in colon tissues, as assessed by qRT‐PCR (V) and Western blot (W). X) mRNA expression levels of TGF‐β and IL‐4 in colon tissues measured by qRT‐PCR. Y) mRNA expression levels of inflammatory cytokines TNF‐ɑ, IL‐6, IL‐10, and IL‐1β in colon tissues measured by qRT‐PCR. (*P < 0.05, **P < 0.01, ***P < 0.001).

Given the high degree of homology between pigs and humans,^[^ ^19^ ^]^ as well as the diverse causes of post‐weaning diarrhea in piglets—ranging from stress and nutritional factors to infections—we selected weaned piglets as the model to investigate how zinc influences gut health. This model closely mirrors key features of human intestinal dysfunction, such as increased permeability and inflammation. To assess the effects of zinc oxide (ZnO) on gut barrier dysfunction, we supplemented the diets of piglets with ZnO for five days, while the control group received a standard diet (**Figure** **1**A). We specifically chose ZnO as the zinc source because of its extremely low solubility in water, which makes it unsuitable for suspension‐based gavage. Instead, ZnO can be uniformly mixed into feed, ensuring consistent daily intake and sustained release of zinc ions throughout the gastrointestinal tract. ZnO supplementation reduced gut inflammation by downregulating the mRNA expression of pro‐inflammatory cytokines (IL‐6, IL‐8, IL‐12, IFN‐γ, TLR4, and TLR2) and upregulating anti‐inflammatory cytokines (IL‐4 and TGF‐β) (Figure 1B,C). In addition, ELISA assays were performed to measure the protein levels of inflammatory cytokines in colon tissues. The results showed that ZnO supplementation significantly decreased the levels of pro‐inflammatory cytokines (TNF‐α, IL‐1β, and IL‐6) and increased the levels of anti‐inflammatory cytokines (IL‐4 and TGF‐β), further confirming its anti‐inflammatory effects (Figure S1A,B, Supporting Information). Additionally, ZnO‐treated piglets had significantly fewer instances of diarrhea, lower fecal scores, and reduced expression of the diarrhea‐associated gene CFTR (Figure S1C–E, Supporting Information). ZnO treatment also increased the mRNA and protein expression of gut barrier genes, such as ZO‐1, occludin, and claudin‐1, which are critical for maintaining gut integrity (Figure 1D,E; Figure S1F–H, Supporting Information). Histological analysis further revealed improved tissue integrity and reduced colon damage in ZnO‐treated piglets (Figure 1F). These findings demonstrate ZnO's potential to reduce gut inflammation and improve gut inflammation and barrier function in weaned piglets.

To understand the mechanisms behind ZnO's effects, transcriptome analysis was conducted on colon tissues from control and ZnO‐treated piglets. Results showed that ZnO activated the PI3K‐AKT signaling pathway (Figure 1G). This was supported by a significant increase in AKT phosphorylation in colon tissues from ZnO‐treated piglets (Figure 1H,I), suggesting that the activation of the PI3K‐AKT pathway contributes to ZnO's protective role in gut barrier function. Further, we evaluated the therapeutic potential of zinc oxide nanoparticles (ZnO NPs) for colitis by administering ZnO NPs orally for six days in a DSS‐induced gut barrier injury model. For a more accurate assessment of zinc's role in regulating gut barrier function, the mice were fed a zinc‐deficient diet, ensuring that their zinc intake came exclusively from the experimental diet. ZnO NPs were specifically chosen due to their superior dispersibility in aqueous solution, which allows for stable suspension and precise dosing through oral administration (Figure 1J). ZnO NPs treatment effectively reduced colon shortening and weight loss in DSS‐induced mice (Figure 1K; Figure S1I, Supporting Information). Histological analysis showed that mice treated with ZnO NPs exhibited preserved colonic structure and significantly reduced tissue damage compared to controls (Figure 1L). ZnO NP treatment enhanced the expression of gut barrier genes in a dose‐dependent manner while reducing gut inflammation (Figure 1M,N). This was evident from decreased expression of pro‐inflammatory cytokines (TNF‐α, IL‐6, and IL‐1β) and anti‐inflammatory cytokines (IL‐4 and TGF‐β) (Figure 1O,P). Consistently, ELISA analysis of colon tissues revealed a reduction in the protein levels of TNF‐α, IL‐6, and IL‐1β, along with elevated levels of IL‐4 and TGF‐β, further supporting the anti‐inflammatory effect of ZnO NPs (Figure S1J,K, Supporting Information). Furthermore, considering the importance of the AKT signaling pathway in the protective effects observed in piglets, we found that ZnO NPs also increased AKT phosphorylation in mouse colon tissues (Figure 1Q). To further confirm the critical role of AKT signaling in zinc‐mediated protection against colitis, we performed an intervention study using the AKT inhibitor MK‐2206 (Figure 1R). The results showed that MK‐2206 markedly attenuated the protective effects of ZnO NPs, as evidenced by shortened colon length, worsened colonic histology, and suppressed AKT activation (Figure 1S–U). In addition, the expression of tight junction proteins, anti‐inflammatory cytokines, and pro‐inflammatory cytokines at both the mRNA and protein levels also demonstrated that MK‐2206 antagonized the beneficial effects of ZnO NPs on intestinal barrier function and inflammation (Figure 1V–Y; Figure S1L,M, Supporting Information).

These findings highlight zinc's ability to mitigate gut injury and inflammation, with the activation of the PI3K‐AKT pathway playing a key role in these protective effects.

### 2.2 Histidine Antagonizes Zinc‐Induced AKT Activation

**Figure 2:**
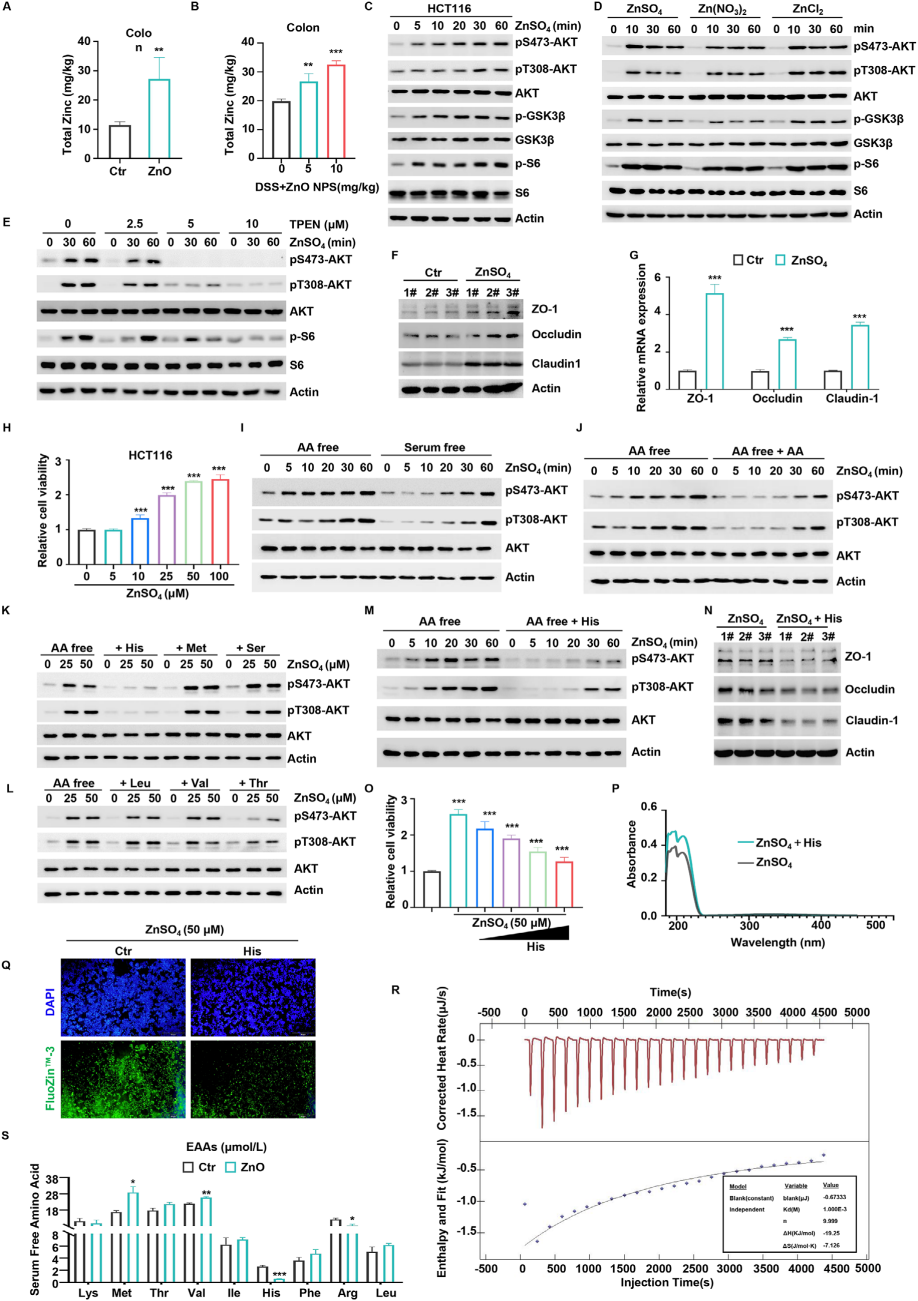
Histidine antagonizes zinc‐induced AKT activation. Total zinc in piglet's colon tissue was detected by flame atomic absorption. B) Total zinc in mice's colon tissue was detected by colorimetric method. C) HCT116 cells were starved in PBS for 1 h, and then supplemented with ZnSO_4_ for the indicated time. The level of pS473‐AKT, pT308‐AKT, p‐GSK3β, p‐S6 and the indicated protein was detected by WB. D) HCT116 cells were starved of PBS for 1 h and then supplemented with ZnSO_4_, Zn(NO_3_)_2_ or ZnCl_2_ for the indicated time. The level of pS473‐AKT, pT308‐AKT, p‐GSK3β, p‐S6 and the indicated protein was detected by WB. E) HCT116 cells were treated with TPEN (0, 2.5, 5, and 10 µm) for 1 h, starved in PBS for 1 h, and then supplemented with ZnSO_4_ for the indicated time. The level of pS473‐AKT, pT308‐AKT, p‐S6 and the indicated protein was detected by WB. F,G) HCT116 cells were starved of PBS for 1 h and then supplemented with ZnSO_4_ for 1 h. The expression levels of ZO‐1, occludin and claudin‐1 in the HCT116 cells were detected by WB (F) or qRT‐PCR (G). H) HCT116 cells were treated with the indicated concentrations of ZnSO_4_ for 48 h. Cell viability was detected using the CCK‐8 assay, n = 3. I) HCT116 cells were starved in serum‐free or amino acid‐free medium for 1 h, and then supplemented with ZnSO_4_ for the indicated time and then supplemented with ZnSO_4_ for 1 h. The level of pS473‐AKT, pT308‐AKT and the indicated protein was detected by WB. J) HCT116 cells were starved in amino acid‐free medium for 1 h, then supplemented with ZnSO_4_ for the indicated time and then supplemented with ZnSO_4_ for 1 h, either alone or combined with amino acids. The level of pS473‐AKT, pT308‐AKT and indicated protein was analyzed by WB. K) HCT116 cells were starved in amino acid‐free medium, then supplemented with ZnSO_4_ in concentration gradients of 0, 25, and 50 µm, either alone or combined with histidine, methionine, or serine for 1 h. The level of pS473‐AKT, pT308‐AKT and indicated protein was analyzed by WB. L) HCT116 cells were starved in amino acid‐free medium for 1 h, then supplemented with ZnSO_4_ in concentration gradients of 0, 25, and 50 µm, either alone or combined with leucine, valine, or threonine for 1 h. The level of pS473‐AKT, pT308‐AKT and indicated protein was analyzed by WB. M) HCT116 cells were starved in amino acid‐free medium for 1 h, then supplemented with 50 µm ZnSO_4_ for 0, 5, 10, 20, 30, or 60 min, either alone or combined with histidine. The level of pS473‐AKT, pT308‐AKT and indicated protein was analyzed by WB. N) HCT116 cells were starved in amino acid‐free medium for 1 h, then supplemented with 50 µµ ZnSO_4_ for 1 h, either alone or combined with histidine. The level of ZO‐1, occludin1, claudin‐1 and indicated protein was analyzed by WB. O) HCT116 cells were treated with the indicated concentrations of ZnSO_4_ and histidine for 48 h. Cell viability was detected using the CCK‐8 assay, n = 3. P) The ZnSO_4_ alone or in the presence of histidine were incubated, and absorbance values were performed in the 185–500 nm wavelength range. Q) HCT116 cells were starved in amino acid‐free medium for 1 h, then supplemented with 50 µµ ZnSO_4_ for 1 h, either alone or combined with histidine. Intracellular labile zinc was measured using FluoZin‐3 AM, with cell nuclei counterstained by DAPI. R) The interaction between ZnSO_4_ and histidine was examined in vitro using isothermal titration calorimetry (ITC). (S). Serum free amino acid in piglet's colon tissue was detected by LC‐MS. (*P < 0.05, **P < 0.01, ***P < 0.001).

To investigate how ZnO affects gut barrier functions, we measured zinc levels in the colons of piglets and mice following ZnO treatment. Zinc content was significantly higher in the treated groups compared to controls (**Figure** **2**A,B), indicating that ZnO primarily acts in the colon as ionic zinc to regulate gut health.

However, the low solubility of ZnO and ZnO NPs presents challenges for consistent and reproducible dosing in vitro studies. Therefore, we employed zinc sulfate, a water‐soluble and bioavailable zinc compound, to precisely control ionic zinc concentrations and exposure durations. We exposed HCT116 and IPEC‐J2 cells to varying concentrations of zinc over different time periods. Zinc treatment resulted in a time‐dependent increase in the phosphorylation of AKT at Ser473 and Thr308, as well as GSK3β phosphorylation (Figure 2C; Figure S2A, Supporting Information). Additionally, zinc enhanced the activity of the downstream mTORC1 pathway, as shown by increased S6 phosphorylation (Figure 2C; Figure S2A, Supporting Information). To further confirm that Zn^2^⁺ is the active form responsible for this effect, we also treated cells with ZnO NPs and observed comparable AKT activation. Given that ZnO NPs release Zn^2^⁺ gradually, these results collectively suggest that zinc activates AKT signaling via its ionic form (Figure S2B, Supporting Information). Moreover, when cells were exposed to higher concentrations of zinc or prolonged treatment durations, we observed that AKT activation was initially enhanced and subsequently diminished, suggesting a typical transient response characteristic of signal transduction pathways. This dynamic regulation supports the notion that zinc functions as a signaling modulator rather than merely a nutritional cofactor (Figure S2C,D, Supporting Information). When cells were treated with different forms of zinc salts, all forms consistently activated AKT across various cell lines, including RKO, SW620, H1299, and HepG2 (Figure 2D, Figure S2E,F, Supporting Information). Furthermore, zinc rescued AKT activation in PBS‐starved conditions in a concentration‐ and time‐dependent manner (Figure S2G,H, Supporting Information), reinforcing its role in AKT signaling. This was further confirmed using the zinc chelator TPEN, which effectively blocked zinc‐induced AKT activation (Figure 2E; Figure S2I,G, Supporting Information). In addition to activating AKT, zinc increased the expression of key gut barrier proteins, including ZO‐1, occludin, and claudin‐1, at both the mRNA and protein levels in HCT116 cells (Figure 2F,G). Zinc also promoted cell proliferation in a concentration‐dependent manner, as demonstrated by the CCK‐8 assay (Figure 2H), underscoring its essential role in maintaining gut integrity.

The AKT pathway is typically activated by growth factors and amino acid signaling. To determine whether zinc activation of AKT relies on these signals, we pre‐treated intestinal cells with serum‐free or amino acid‐free media before zinc stimulation. Zinc activated the AKT pathway even in the absence of both serum and amino acids. Interestingly, we found that higher zinc concentrations or longer treatment durations were required under serum‐free conditions compared to amino acid‐free conditions, suggesting that amino acids may inhibit zinc‐mediated AKT activation (Figure 2I; Figure S2K,L, Supporting Information). Consistent with this, supplementation of amino acid‐free media with amino acids suppressed zinc‐induced AKT activation (Figure 2J; Figure S2M,N, Supporting Information).

Further analysis identified histidine as a specific amino acid that significantly inhibited zinc‐induced AKT activation (Figure 2K,L; Figure S2O,P, Supporting Information). Histidine also reduced the expression of gut barrier proteins ZO‐1, occludin, and claudin‐1 (Figure 2N; Figure S2Q, Supporting Information) and counteracted the zinc‐induced enhancement of cell proliferation (Figure 2O). The chelating properties of histidine were analyzed using UV‐visible spectroscopy. Adding histidine to ZnSO₄ induced a distinct optical shift, indicating the formation of a zinc–histidine chelate complex (Figure 2P). In live cells, zinc fluorescence intensity detected by FluoZin‐3 AM significantly decreased following histidine treatment, further confirming that histidine effectively chelates intracellular zinc (Figure 2Q). To provide direct evidence of this interaction, we performed isothermal titration calorimetry (ITC), which demonstrated a strong binding affinity between zinc and histidine (Figure 2R). Furthermore, to investigate the in vivo relevance of this interaction, we measured amino acid profiles in colon tissues of ZnO‐treated piglets and observed a significant reduction in histidine levels compared to controls (Figure 2S), suggesting a potential regulatory relationship between zinc supplementation and histidine bioavailability.

### 2.3 Promotion of AKT Activation by Zinc through the ZNG1‐METAP1 Axis

Next, we tried to identify the proteins responsible for the activation of the AKT pathway by zinc. Previous studies have shown that GPR39, ERK, and ZIP1 are involved in regulating intracellular signaling pathways.^[^ ^9a,c,d^ ^]^ However, neither the ERK inhibitor SCH772984 nor knockdown of GPR39 or ZIP1 affected the zinc‐induced AKT activation (Figure S3A–C, Supporting Information). Thus, the activation of the AKT pathway by zinc was not dependent on GPR39, ERK, or ZIP1.

**Figure 3:**
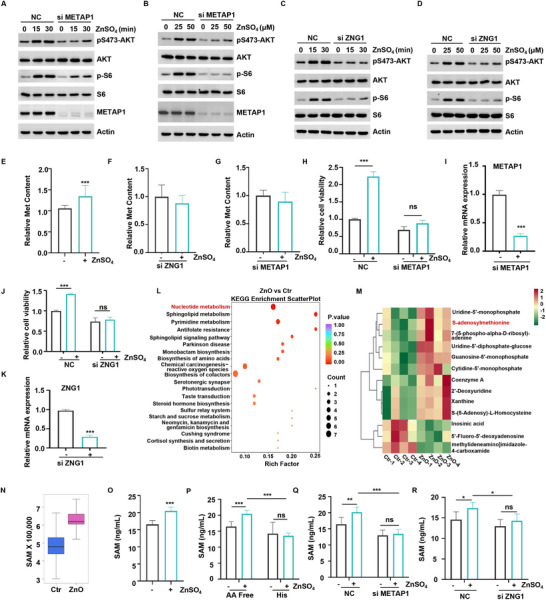
Promotion of AKT activation by zinc through the ZNG1‐METAP1 axis. A,B) METAP1 WT or knockdown HCT116 cells were starved in amino acid‐free medium for 1 h and then supplemented with ZnSO_4_ at the indicated concentration (A) or the indicated time (B). The level of pS473‐AKT, p‐S6, and the indicated protein was detected by WB. C,D) ZNG1 WT or knockdown HCT116 cells were starved in amino acid‐free medium for 1 h and then supplemented with ZnSO_4_ at the indicated concentration (C) or the indicated time (D). The level of pS473‐AKT, p‐S6, and the indicated protein was detected by WB. E) HCT116 cells were starved in amino acid‐free medium and then supplemented with 50 µm ZnSO_4_ for 1 h. The intracellular Met levels were assayed by LC‐MS. F,G) ZNG1 (F) and METAP1 (G) WT or knockdown HCT116 cells were starved in amino acid‐free medium and then supplemented with 50 µM ZnSO_4_ for 1 h. The intracellular Met levels were assayed by LC‐MS. H–K). METAP1 (H), and ZNG1 (J) WT or knockdown HCT116 cells were treated with of 50 µm ZnSO_4_ for 48 h. Cell viability was detected using the CCK‐8 assay, n = 3. The knockdown efficiency of METAP1 (I), and ZNG1 (K) was shown. (L). KEGG enrichment scatter plot for colonic metabolites in piglets from the control group and ZnO treatment group. M) Metabolite enrichment map for nucleotide metabolism pathway in colonic metabolites of piglets from the control group and ZnO treatment group. N) SAM levels of the control group and ZnO treatment group. O) HCT116 cells were starved in amino acid‐free medium for 1 h, then supplemented with 50 µµ ZnSO_4_ for 1 h. The intracellular SAM levels were detected by Elisa. P) HCT116 cells were starved in amino acid‐free medium for 1 h, then supplemented with 50 µµ ZnSO_4_ for 1 h, either alone or combined with histidine. The intracellular SAM levels were detected by Elisa. Q,R) METAP1 (Q) and ZNG1 (R) WT or knockdown HCT116 cells were starved in amino acid‐free medium and then supplemented with 50 µm ZnSO_4_ for 1 h. The intracellular SAM levels were detected by Elisa. (*P < 0.05, **P < 0.01, ***P < 0.001).

A recent study revealed that ZNG1 functions as a zinc metallochaperone and transfers zinc to METAP1, thereby activating its enzymatic activity.^[^ ^20^ ^]^ Therefore, we investigated whether ZNG1 and METAP1 are involved in the regulation of zinc‐mediated activation of AKT. On knocking down ZNG1 and METAP1, we found that the absence of ZNG1 and METAP1 blocked the activation of AKT by zinc (**Figure** **3**A–D; Figure S3D–G, Supporting Information). This result suggests that ZNG1 and METAP1 play a role in the zinc‐induced activation of the AKT pathway.

Given that the primary function of METAP1 is to remove the initiating methionine residue from newly synthesized polypeptide chains, we tried to determine whether zinc treatment leads to an increase in intracellular methionine levels. Our findings revealed that methionine concentrations in the intestines of piglets from the ZnO‐treated group were significantly higher than those in the control group piglets (Figure 2R). Further validation using liquid chromatography‐mass spectrometry demonstrated a significant elevation in intracellular methionine levels after zinc treatment that was abrogated on knocking down ZNG1 and METAP1 (Figure 3E–G). To further investigate whether the ZNG1‐METAP1 axis plays a critical role in zinc‐mediated regulation of cell proliferation, we performed knockdown experiments targeting these proteins in HCT116 and IPEC‐J2 cells. Our results demonstrated that the proliferative effects of zinc were completely abrogated following the knockdown of ZNG1 and METAP1 (Figure 3H–K; Figure S3H–K, Supporting Information). These findings indicate that ZNG1 and METAP1 are indispensable for zinc‐induced AKT activation and subsequent cell proliferation.

To further identify the metabolites that control zinc‐mediated AKT activation, we performed untargeted metabolomic sequencing of gut tissues (Figure S3L, Supporting Information). We identified 200 differentially expressed metabolites, including 105 upregulated and 95 downregulated metabolites (Figure S3M, Supporting Information). KEGG enrichment analysis revealed that the major metabolic pathways affected by ZnO treatment included nucleotide metabolism, sphingolipid metabolism, pyrimidine metabolism, antifolate resistance, and sphingolipid signaling pathways (Figure 3L). Given the significant enrichment of the nucleotide metabolism pathway, we further analyzed the differentially expressed metabolites within this pathway and found significant upregulation of SAM in the ZnO‐treated group (Figure 3M,N). Validation analysis using an ELISA kit confirmed that the intracellular SAM levels were significantly elevated after zinc treatment (Figure 3O; Figure S3N, Supporting Information) and were reduced in response to ZNG1 and METAP1 knockdown (Figure S3O–T, Supporting Information). Accordingly, the knockdown of ZNG1 or METAP1 blocked the zinc‐induced increase in SAM levels, and a similar effect was observed with the addition of histidine (Figure 3P–R; Figure S3U–Y, Supporting Information). This suggests that the zinc/ZNG1‐METAP1 axis regulates intracellular SAM levels. Taken together, the findings of the experiments described in this subsection point to the role of the ZNG1‐METAP1‐SAM axis in the zinc‐induced activation of the AKT pathway and consequent alleviation of gut barrier dysfunction.

### 2.4 Protective Effect of Zinc on the Gut Barrier through PRMT5‐Mediated Methylation of AKT

**Figure 4:**
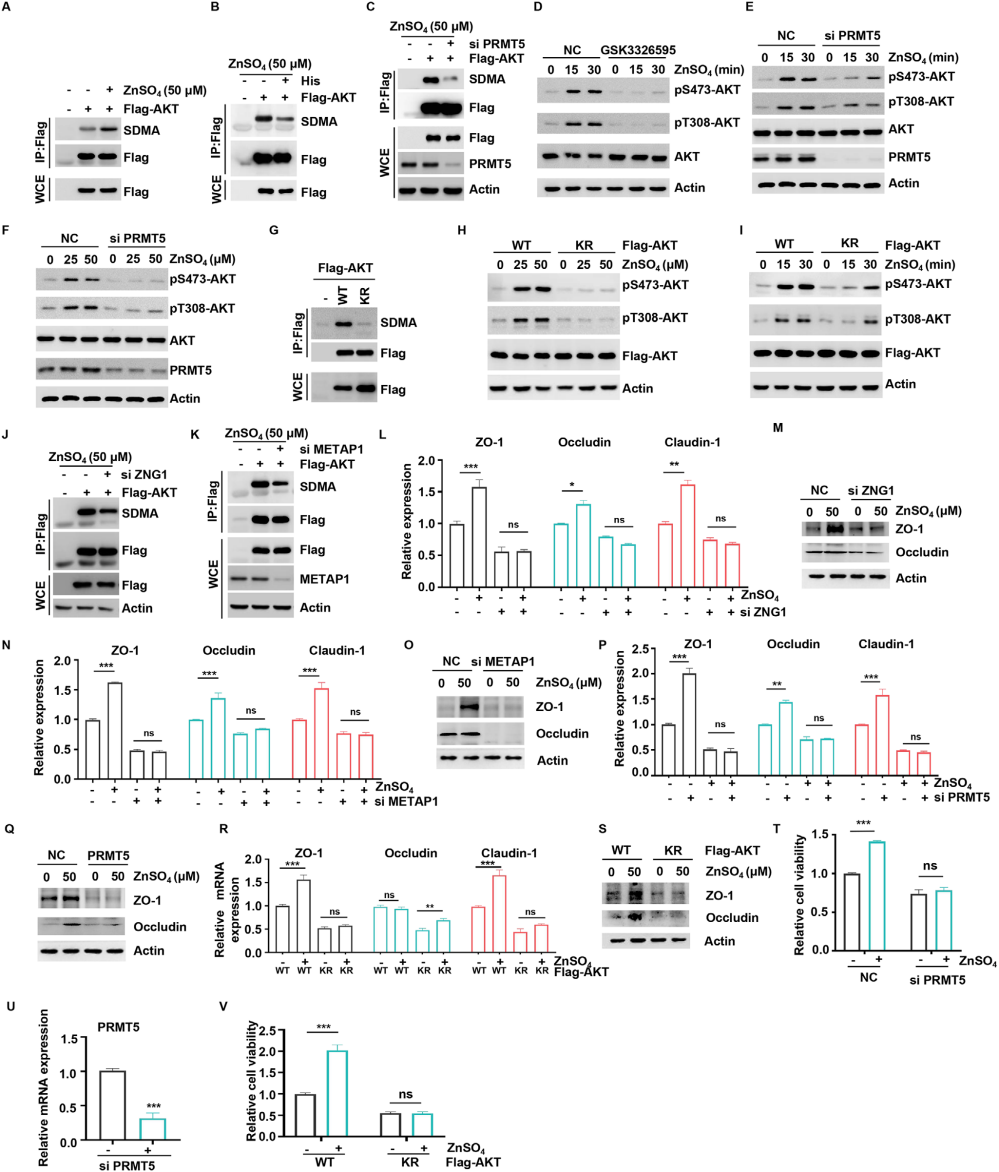
Protective effect of zinc on the intestinal barrier through PRMT5‐mediated methylation of AKT. A,B) HEK293T cells were transfected with Flag‐AKT. After transfection for 24 h, cells were starved in amino acid‐free medium for 1 h, then supplemented with 50 µm ZnSO_4_ for 1 h, either alone (A) or combined with histidine (B). AKT was immunoprecipitated and subsequently analyzed by WB for symmetric dimethylarginine (SDMA) modification. C) PRMT5 WT or knockdown HEK293T cells were starved in amino acid‐free medium for 1 h, then supplemented with 50 µm ZnSO_4_ for 1 h. AKT was immunoprecipitated and subsequently analyzed by WB for SDMA modification. D) HCT116 cells were treated with GSK3326595 (10 µm) for 6 h, HCT116 cells were starved in amino acid‐free medium for 1 h, and then supplemented with ZnSO_4_ for the indicated time. The level of pS473‐AKT, pT308‐AKT and indicated protein was analyzed by WB. E,F) PRMT5 WT or knockdown HCT116 cells were starved in amino acid‐free medium for 1 h and then supplemented with ZnSO_4_ at the indicated concentration (E) or the indicated time F) The level of pS473‐AKT, pT308‐AKT and indicated protein was analyzed by WB. G) Flag‐AKT‐WT and Flag‐AKT‐KR‐overexpressing HEK293T cells were starved in amino acid‐free medium for 1 h and then supplemented with ZnSO_4_ for 1 h. AKT was immunoprecipitated and subsequently analyzed by WB for SDMA modification. H,I) Flag‐AKT‐WT and Flag‐AKT‐KR‐overexpressing HEK293T cells were starved in amino acid‐free medium for 1 h and then supplemented with ZnSO_4_ at the indicated concentration (H) or the indicated time (I). The level of pS473‐AKT, pT308‐AKT and indicated protein was analyzed by WB. J,K) METAP1 (J) and ZNG1 (K) WT or knockdown HEK293T cells were starved in amino acid‐free medium and then supplemented with 50 µM ZnSO_4_ for 1 h. AKT was immunoprecipitated and subsequently analyzed by WB for SDMA modification. L–Q) ZNG1 (L, M), METAP1 (N, O), and PRMT5 (P, Q) WT or knockdown HCT116 cells were starved in amino acid‐free medium for 1 h and then supplemented with 50 µm ZnSO_4_ for 1 h. The expression levels of ZO‐1, occludin, and claudin‐1 were detected by qRT‐PCR (L, N, P) or WB (M, O, Q). R,S). Flag‐AKT‐WT and Flag‐AKT‐KR‐overexpressing HCT116 cells were starved in amino acid‐free medium for 1 h and then supplemented with 50 µm ZnSO_4_ for 1 h. The expression levels of ZO‐1, occludin and claudin‐1were detected by qRT‐PCR (R) or WB (S). T,U). PRMT5 WT or knockdown HCT116 cells were treated with of 50 µm ZnSO_4_ for 48 h. Cell viability was detected using the CCK‐8 assay, n = 3. The knockdown efficiency of PRMT5 (U) was shown. V) Flag‐AKT‐WT and Flag‐AKT‐KR‐overexpressing HCT116 cells were treated with of 50 µm ZnSO_4_ for 48 h. Cell viability was detected using the CCK‐8 assay, n = 3. (*P < 0.05, **P < 0.01, ***P < 0.001).

SAM, as a methyl donor, facilitates the covalent methylation of target proteins through the action of specific enzymes.^[^ ^21^ ^]^ Several studies have reported that PRMT5 specifically mediates AKT methylation modification, thereby activating the AKT pathway.^[^ ^18^ ^]^ Here, we have investigated whether zinc promotes methylation of AKT. To this end, intracellular AKT was immunoprecipitated and incubated with an SDMA pan‐antibody. PRMT5 is responsible for catalyzing the symmetrical dimethylation of arginine residues in proteins, a process known as symmetrical dimethylarginine (SDMA).^[^ ^22^ ^]^ Our results demonstrated that zinc could enhance AKT methylation (**Figure** **4**A), which was attenuated by histidine supplementation (Figure 4B). However, zinc‐mediated AKT methylation levels were impaired by PRMT5 knockdown or the presence of PRMT5 inhibitors (Figure 4C; Figure S4A, Supporting Information). Similarly, both knockdown of PRMT5 and pharmacological inhibition using its specific inhibitor GSK3326595 resulted in a reduction in zinc‐mediated AKT phosphorylation (Figure 4D–F; Figure S4B–D, Supporting Information).

It has been documented that methylation of Arg15 and Arg391 is essential for the activation of AKT by PRMT5.^[^ ^18^ ^]^ Therefore, we aimed to ascertain whether PRMT5‐mediated R15/R391 methylation contributes to zinc‐activated AKT activation. To this end, we generated a mutant form of Flag‐AKT‐KR (R15K/R391K). Overexpression of Flag‐AKT‐KR inhibited intracellular AKT methylation (Figure 4G). Furthermore, the AKT‐KR mutation abrogated the effect of zinc on AKT phosphorylation (Figure 4H,I). These results indicate that PRMT5‐mediated AKT methylation is indispensable for zinc‐induced activation of AKT.

In our previous experiments, we observed that zinc, in conjunction with ZNG1 and METAP1, results in elevated intracellular SAM levels, thereby activating AKT. Consequently, we further investigated the involvement of ZNG1 and METAP1 in zinc‐mediated enhancement of AKT methylation levels. Our findings indicated that knockdown of METAP1 and ZNG1 can impede AKT methylation (Figure 4J,K; Figure S4E, Supporting Information). Importantly, in the absence of endogenous METAP1, ZNG1, or PRMT5, the mRNA and protein levels of the intestinal barrier genes ZO‐1, Occludin, and Claudin‐1 were significantly reduced (Figure S4F–K, Supporting Information). Notably, even upon zinc stimulation, their expression could not be restored or upregulated (Figure 4L–Q). This suggests that the ZNG1–METAP1–PRMT5 axis is a necessary upstream mediator for AKT methylation and subsequent barrier gene expression in response to zinc. Notably, overexpression of Flag‐AKT‐KR effectively inhibited the promotive effects of zinc on the gut barrier‐related genes ZO‐1, occludin, and claudin‐1 (Figure 4R,S). To further validate whether the ZNG1‐METAP1‐PRMT5 axis is involved in zinc‐mediated regulation of cell proliferation, we knocked down these proteins in HCT116 and IPEC‐J2 cells and found that the effect of zinc on cell proliferation was completely abolished (Figure 4T,U; Figure S4L,M, Supporting Information). Moreover, compared to wild‐type AKT, overexpression of the AKT‐KR mutant led to a significant reduction in cell proliferation, and the stimulation of cell proliferation by zinc was significantly eliminated (Figure 4V). This finding suggests that AKT methylation plays a crucial role in the promotion of cell proliferation by zinc. The findings described above imply that AKT methylation plays an important role in gut health and is essential for the alleviate gut barrier dysfunction effects of zinc supplementation.

### 2.5 Promotion of AKT Cell Membrane Localization by Zinc via AKT Methylation

**Figure 5:**
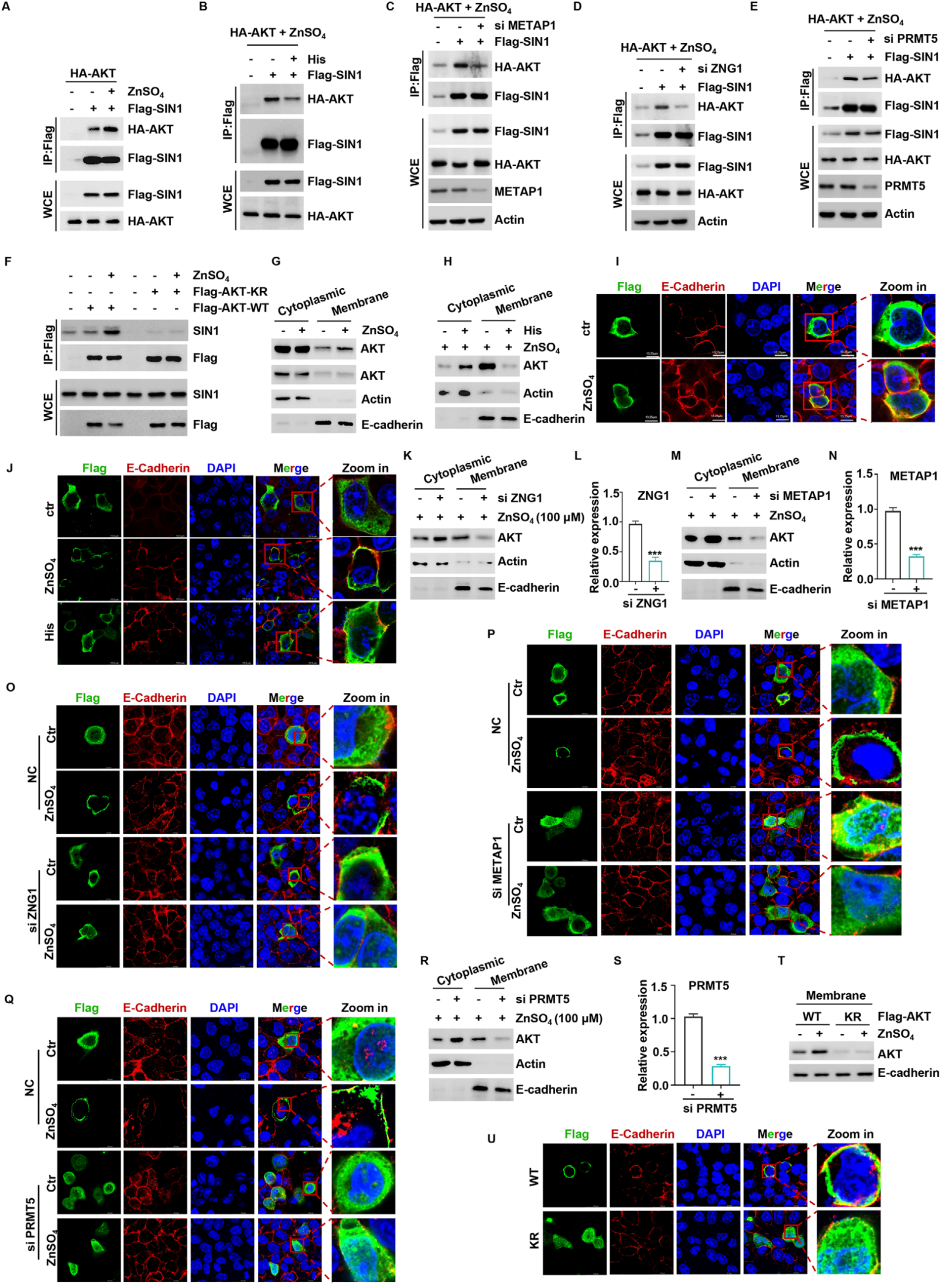
Zinc promotes AKT cell membrane localization via AKT methylation. A). HEK293T cells were starved in amino acid‐free medium for 1 h and then supplemented with 50 µm ZnSO_4_ for 1 h, either alone (A) or combined with histidine B) The interaction of HA‐AKT and Flag‐SIN1 were analyzed by Co‐IP assay. C–E) METAP1 (C), ZNG1 (D), and PRMT5 (E) WT or knockdown HEK293T cells were starved in amino acid‐free medium for 1 h and then supplemented with 50 µM ZnSO_4_ for 1 h. The interaction of HA‐AKT and Flag‐SIN1 were analyzed by Co‐IP assay. F) Flag‐AKT‐WT and Flag‐AKT‐KR‐overexpressing HEK293T cells were starved in amino acid‐free medium for 1 h and then supplemented with 50 µM ZnSO_4_ for 1 h. The interaction of HA‐AKT and Flag‐SIN1 were analyzed by Co‐IP assay. G–J) HEK293T cells were starved in amino acid–free medium for 1 h, followed by treatment with 50 µm ZnSO_4_ for 1 h, either alone (G, I) or in combination with histidine (H, J). Subcellular protein fractionation was analyzed by WB (G, H). The subcellular localization of AKT in HCT116 cells was further assessed by IF, with E‐Cadherin used as a plasma membrane marker (I, J). ZNG1 (K), METAP1 (M) and PRMT5 (R) WT or knockdown HEK293T cells were starved in amino acid‐free medium for 1 h and then supplemented with 50 µm ZnSO_4_ for 1 h. Subcellular protein fractionation was analyzed by WB. (O‐Q). The subcellular localization of AKT in HCT116 cells was further assessed by IF, with E‐Cadherin used as a plasma membrane marker. (T). Flag‐AKT‐WT and Flag‐AKT‐KR‐overexpressing HEK293T cells were starved in amino acid‐free medium for 1 h and then supplemented with 50 µM ZnSO_4_ for 1 h. Subcellular protein fractionation was analyzed by WB. U) The subcellular localization of AKT in HCT116 cells was further assessed by IF, with E‐Cadherin used as a plasma membrane marker. (*P < 0.05, **P < 0.01, ***P < 0.001).

Previous research has underscored the pivotal function of PRMT5‐mediated AKT methylation in AKT plasma membrane localization and its interaction with mTORC2.^[^ ^18a^ ^]^ Therefore, we delved deeper into the influence of zinc on these biological processes. Initially, we assessed the impact of zinc on the interaction between AKT and the mTORC2 component protein SIN1. Co‐immunoprecipitation experiments revealed that zinc significantly potentiated the binding of SIN1 to AKT, and this enhancement was abrogated by the addition of histidine (**Figure** **5**A,B). Further, our findings indicated that the knockdown of METAP1 and ZNG1 abrogated zinc‐mediated AKT‐SIN1 binding (Figure 5C,D). We also observed that the enhanced interaction between zinc‐mediated AKT and SIN1 was impaired under conditions of PRMT5 depletion (Figure 5E). Finally, we investigated whether PRMT5‐mediated methylation of AKT1 at Arg15 and Arg391 is required for zinc‐mediated protein–protein interactions. Our results demonstrated that Flag‐AKT‐KR, a mutant form of AKT, failed to bind to SIN1, even under conditions of zinc stimulation (Figure 5F).

To determine whether zinc promotes the localization of AKT to the plasma membrane, we first performed cell fractionation assays and found that the level of AKT in the membrane fraction increased following zinc treatment (Figure 5G). Conversely, histidine‐treated cells exhibited a marked reduction in membrane‐localized AKT (Figure 5H). To further validate these findings, we overexpressed Flag – AKT in HCT116 cells and visualized its subcellular distribution by immunofluorescence (IF). Zinc treatment significantly enhanced AKT localization at the plasma membrane, consistent with the fractionation results (Figure 5I). In contrast, co‐treatment with histidine markedly impaired zinc‐induced membrane recruitment of AKT, resulting in diffuse cytoplasmic distribution (Figure 5J). These results further support that histidine disrupts zinc‐mediated AKT translocation by chelating intracellular zinc, thereby blocking its activation and downstream signaling. Additionally, knockdown of METAP1, ZNG1, or PRMT5 by siRNA suppressed zinc‐mediated AKT localization to the plasma membrane, as shown by both membrane fractionation and IF staining (Figure 5K–S). Similarly, the AKT‐KR methylation‐deficient mutant failed to localize to the plasma membrane regardless of zinc stimulation, as demonstrated by cell fractionation and IF analysis (Figure 5T,U). These results indicate that zinc promotes AKT membrane localization in a manner that requires PRMT5‐mediated methylation, and disruption of this modification—either genetically or via pathway interference—blocks zinc's effect on AKT activation.

### 2.6 Impact of Histidine on Zinc Efficacy in Alleviating Barrier Dysfunction

**Figure 6:**
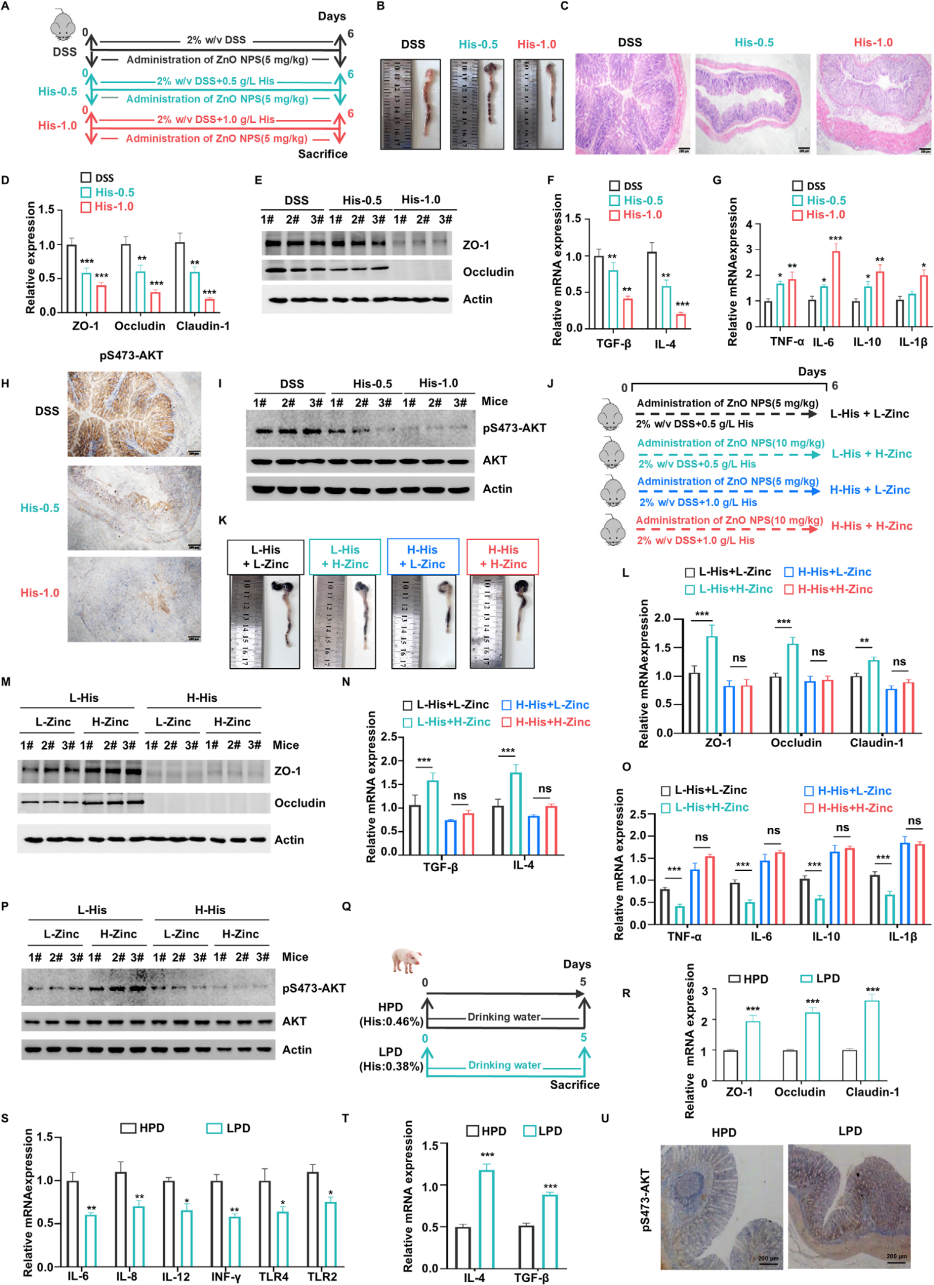
Impact of histidine on zinc efficacy in reducing intestinal inflammation and barrier dysfunction. A) Flowchart of Experiment on mice. During the experiment, all mice were provided with drinking water treated with 2% DSS. Group 1 (DSS group) continued to receive DSS water as provided. Meanwhile, Group 2 (His‐0.5) and Group 3 (His‐1.0) had their drinking water supplemented with histidine at concentrations of 0.5 and 1.0 g L^−1^, respectively, to investigate the potential antagonistic effects of histidine on ZnO NPs. B) Colon length of the DSS group, His‐0.5 group and His‐1.0 group. C) H&E staining of colon tissue in piglets of the DSS group, His‐0.5 group and His‐1.0 group. D,E) The expression levels of ZO‐1, occludin and claudin‐1 in the colon tissue of mice were detected by qRT‐PCR (D) or WB (E). F) The expression levels of TGF‐β and IL‐4 in the colon tissue of mice were detected by qRT‐PCR. G) The expression levels of TNF‐α, IL‐6, IL‐10, and IL‐1β in the colon tissue of mice were detected by qRT‐PCR. H,I) AKT activity in mice colon tissue was detected by immunohistochemical staining (H) or WB (I). J) Flowchart of Experiment on mice. All mice were fed a diet deficient in both histidine and zinc and provided with 2% DSS in drinking water throughout the experiment. The four groups in the 2 × 2 factorial design were as follows: Group 1: Low Histidine (L‐His) + Low Zinc (L‐Zinc); Group 2: Low Histidine (L‐His) + High Zinc (H‐Zinc); Group 3: High Histidine (H‐His) + Low Zinc (L‐Zinc); Group 4: High Histidine (H‐His) + High Zinc (H‐Zinc). K) Colon length of the L‐His + L‐Zinc group, L‐His + H‐Zinc group, H‐His + L‐Zinc, and H‐His + H‐Zinc group. L,M) The expression levels of ZO‐1, occludin and claudin‐1 in the colon tissue of mice were detected by qRT‐PCR (L) or WB (M). N) The expression levels of TGF‐β and IL‐4 in the colon tissue of mice were detected by qRT‐PCR. O) The expression levels of TNF‐α, IL‐6, IL‐10, and IL‐1β in the colon tissue of mice were detected by qRT‐PCR. P) AKT activity in mice colon tissue was detected by immunohistochemical staining WB. Q) Experimental flowchart for piglet. For 5 days, the HPD group (n = 8) received a diet containing 20% protein, whereas the LPD group (n = 8) was administered a diet comprising 17% protein. R) The expression levels of ZO‐1, occludin and claudin‐1 in the colon tissue of piglets from the control group and ZnO treatment group were detected by qRT‐PCR. S) The expression levels of IL‐6, IL‐8, IL‐12, INF‐γ, TLR2, and TLR4 in the colon tissue of piglets from the control group and ZnO treatment group were detected by qRT‐PCR. T) The expression levels of IL‐4 and TGF‐β in the colon tissue of piglets from the control group and ZnO treatment group were detected by qRT‐PCR. (U). AKT activity in piglet colon tissue was detected by immunohistochemical staining. (*P<0.05, **P<0.01, ***P<0.001).

We investigated the antagonistic effect of histidine on zinc‐mediated improvements in gut barrier function using a DSS‐induced gut barrier injury model in mice. To accurately assess histidine's impact, a histidine‐deficient diet was provided, ensuring its intake was independent of variations in food consumption (**Figure** **6**A). The results showed that when histidine was administered alongside ZnO NPs at various concentrations, histidine dose‐dependently worsened DSS‐induced colon shortening and damage to colonic microvilli (Figure 6B,C). Histidine supplementation also suppressed the expression of gut barrier‐related genes and increased the upregulation of pro‐inflammatory cytokines while decreasing anti‐inflammatory cytokines (Figure 6D–G; Figure S5A,B, Supporting Information). In addition, histidine inhibited Akt phosphorylation, further undermining the protective effects of ZnO NPs (Figure 6H,I). These findings highlight that histidine counteracts zinc's beneficial effects, exacerbating gut injury and inflammation.

To further investigate the interaction between histidine and zinc, we conducted a two‐factor crossover trial. Mice were divided into four groups: low histidine with low zinc (L‐His + L‐Zinc), low histidine with high zinc (L‐His + H‐Zinc), high histidine with low zinc (H‐His + L‐Zinc), and high histidine with high zinc (H‐His + H‐Zinc) (Figure 6J). During the trial, the mice were fed a diet deficient in both histidine and zinc, ensuring that their intake of these nutrients depended solely on the amounts added externally. At low histidine concentrations, ZnO NPs retained their anti‐diarrheal effects, alleviating DSS‐induced colon shortening (Figure 6K). Similarly, ZnO NPs at low histidine levels promoted the expression of gut barrier and anti‐inflammatory genes while suppressing pro‐inflammatory genes. However, these beneficial effects were diminished at higher histidine concentrations (Figure 6L–O; Figure S5C,D, Supporting Information). High histidine levels also antagonized ZnO NP‐induced Akt phosphorylation (Figure 6P), highlighting histidine's inhibitory role in zinc‐mediated gut protection.

Next, we investigated the feasibility of a dietary intervention with histidine intake to improve gut barrier dysfunction. Piglets were fed either a low‐protein diet (LPD, low histidine levels, His: 0.38%) or a high‐protein diet (HPD, high histidine levels, His: 0.46%) (Figure 6Q; Figure S5E, Supporting Information). Tight junction protein expression, including ZO‐1, occludin, and claudin‐1, was significantly higher in the LPD group compared to the HPD group (Figure 6R; Figure S5F–I, Supporting Information). The LPD group also exhibited reduced gut inflammation, characterized by decreased levels of pro‐inflammatory cytokines (IL‐6, IL‐8, IL‐12, IFN‐γ, TLR4, and TLR2) and increased anti‐inflammatory cytokines (IL‐4 and TGF‐β) (Figure 6S,T). Consistent with the transcriptional changes, ELISA analysis of colon tissues further confirmed a significant reduction in the protein levels of inflammatory cytokines, supporting the anti‐inflammatory effect of the LPD intervention (Figure S5J,K, Supporting Information). Furthermore, the LPD group experienced a lower incidence of diarrhea, improved fecal scores, less severe colonic damage, and reduced CFTR expression compared to the HPD group (Figure S5F–I, Supporting Information). Enhanced Akt activation was observed in the LPD group, suggesting a mechanistic link between reduced histidine intake and improved gut barrier function (Figure 6U; Figure S5J, Supporting Information).

Finally, we analyzed the amino acid profiles in the two dietary groups to identify factors affecting Akt activity. These findings suggest that a low‐protein diet, by reducing histidine levels, promotes zinc‐mediated activation of the Akt pathway, leading to improved gut barrier integrity and reduced gut inflammation.

## 3 Discussion

In the current study, we found that zinc plays a beneficial role in gut barrier dysfunction. Further exploring the mechanism underlying the beneficial effects of zinc in the management of gut barrier dysfunction, we found that zinc promotes AKT activation independently of its receptor GPR39 and transporter ZIP. Further, the ZNG‐METAP1 signaling axis appears to play a crucial role in zinc‐mediated AKT activation by mediating AKT methylation. Interestingly, HPD seems to suppress the beneficial effects of zinc on the gut barrier. Actually, zinc‐mediated AKT activation could be blocked by histidine. Given the significant role of zinc in alleviating gut barrier dysfunction, our findings are of great importance for the treatment of bowel diseases such as Crohn's disease and Ulcerative colitis.

In recent years, an increasing number of studies have indicated that zinc supplementation plays a pivotal role in the management of gut barrier dysfunction and pediatric diarrhea.^[^ ^23^ ^]^ Concurrently, serum zinc levels may serve as a predictive indicator for assessing the severity of gut barrier dysfunction and diarrhea.^[^ ^24^ ^]^ Studies have reported that children with dehydration and rotavirus diarrhea, as well as patients with irritable bowel syndrome, often exhibit lower serum zinc levels.^[^ ^25^ ^]^ Although further clinical validation is needed, these findings suggest that a reduction in serum zinc levels may be a critical factor contributing to gut barrier dysfunction. Our study used weaned piglets as an animal model and ZnO as the zinc source for treatment, and our results showed that zinc levels in the colon of piglets in the ZnO treatment group were elevated, indicating that zinc levels may be a key factor in the protective effect of zinc on the gut. In accordance with our findings, recent studies have shown that low serum zinc levels (< 0.7 mg/L) at weaning may be a predisposing factor for gut barrier dysfunction in piglets,^[^ ^26^ ^]^ and supplementation with high doses of ZnO can restore serum zinc levels to normal and alleviate gut barrier dysfunction. However, contrary to the use of ZnO as a supplement in weaned piglets to alleviate gut barrier dysfunction and prevent diarrhea, it is not recommended for the treatment of pediatric diarrhea, and this is because ZnO absorption in humans is relatively poor in the absence of food intake.^[^ ^27^ ^]^ Instead, the World Health Organization recommends the use of the zinc supplements ZnSO_4_, (CH_3_COO)_2 _Zn, and C₁₂H₂₂O₁₄Zn in pediatric patients due to their improved absorption.^[^ ^28^ ^]^

While our current study focuses on the short‐term effects of zinc supplementation, we acknowledge the importance of evaluating potential risks associated with long‐term or high‐dose zinc intake. Although prolonged zinc supplementation may enhance epithelial integrity, immune defense, and antioxidant function, excessive zinc intake can inhibit copper absorption, leading to secondary copper deficiency and associated complications such as anemia and immune dysfunction.^[^ ^29^ ^]^ Moreover, high doses of zinc may disturb the gut microbiota composition, contributing to dysbiosis and impaired metabolic homeostasis.^[^ ^30^ ^]^ Therefore, future clinical applications of zinc supplementation should carefully consider dosage and duration to balance its beneficial and adverse effects.

Recent studies have also highlighted that AKT is subject to multiple post‐translational modifications (PTMs), including phosphorylation, ubiquitination, and methylation, which often function in a coordinated manner to regulate its activity, stability, and subcellular localization. These modifications often function in a coordinated and context‐dependent manner. For instance, SETDB1‐mediated methylation at Lys64 has been shown to enhance AKT phosphorylation at key regulatory sites Thr308 and Ser473 and promote oncogenic signaling.^[^ ^17a^ ^]^ Conversely, in certain contexts, methylation can generate recognition motifs for specific E3 ubiquitin ligases, facilitating AKT degradation.^[^ ^31^ ^]^

Multiple studies have demonstrated that AKT methylation probably participates in modulating its activation status and downstream signaling cascades.^[^ ^17a^ ^]^ Our study further expands on these findings by identifying zinc as a critical upstream regulator of AKT methylation in intestinal epithelial cells. Therefore, we explored AKT methylation as a potential mechanism via which zinc induces it activation. We observed that zinc facilitates AKT activation through promoting its methylation modification, thereby activating the AKT pathway. Furthermore, our findings revealed a pivotal role of zinc in regulating the localization of AKT to the plasma membrane through modulating the interaction between AKT and SIN1. Notably, we found that methylation at the R15 and R391 sites of AKT is imperative for the activation of AKT by zinc. Remarkably, mutating both sites to lysine completely nullified the stimulatory effect of zinc on AKT activation. It has been reported that PRMT5‐mediated methylation of AKT weakens interdomain interactions between the PH and KD domains of AKT, leading to a conformational change and thereby promoting AKT kinase activity.^[^ ^18b^ ^]^ However, it remains to be fully elucidated whether zinc influences conformational changes in AKT. Our data also show that AKT methylation plays an important role in zinc‐promoted cell proliferation, but whether it affects zinc‐mediated regulation of gut barrier dysfunction at the in vivo level remains to be further verified through the construction of knock‐in mice.

Beyond its traditional role as a catalytic and structural cofactor, zinc is well known as a signal transducer.^[^ ^32^ ^]^ Five previous studies have established a link between Ras and zinc. Two of these studies found that acute processing of zinc resulted in activation of the EGFR‐Ras‐MEK‐ERK pathway,^[^ ^33^ ^]^ while two studies working with *Caenorhabditis elegans* conducted genetic manipulation experiments and found that zinc antagonized Ras‐MEK‐ERK pathway signaling.^[^ ^34^ ^]^ A study by Anson et al. concluded that Ras is involved in zinc activation of ERK and AKT.^[^ ^13c^ ^]^ These five studies suggest that zinc plays an important role in regulating the Ras‐MEK‐ERK pathway, under both acute (cellular) and chronic (genetic) conditions. However, we found that zinc‐induced activation of AKT is not dependent on ERK, because it was not affected on treatment of HCT116 cells with an inhibitor of ERK. However, we do not have any data demonstrating whether zinc activation of AKT is dependent on Ras, and this needs to be studied more thoroughly in the future. Additionally, zinc has also been reported to have insulin‐like functionality and to independently promote phosphorylation of multiple substrates including RTK, ERK1/2, AKT, and p38.^[^ ^35^ ^]^ However, our results showed that epidermal growth factor receptor (EGFR) inhibition had no effect on zinc‐mediated AKT activation (data not shown). Alternatively, it is possible that this could differ between cell types or model systems, so the role of EGFR is not entirely clear yet and needs to be further investigated. Although we have shown that the HCT116 cell line exhibits zinc‐mediated activation of AKT independent of ERK and EGFR, much remains to be learned about whether different cell systems respond to zinc in unique ways.

Recently, a study published in *Cell* revealed that ZNG1 serves as a zinc chaperone, delivering zinc to apo‐Map1p in a GTPase‐dependent manner to activate the biological function of METAP1.^[^ ^20^ ^]^ METAP1 plays a vital role in protein synthesis and maturation, specifically by removing the N‐terminal methionine from nascent proteins, which is a crucial step in protein biogenesis.^[^ ^11a^ ^]^ Similarly, Dummitt et al. demonstrated that METAP1 is crucial in maintaining methionine homeostasis in *Saccharomyces cerevisiae*, and its absence leads to elevated methionine levels.^[^ ^36^ ^]^ In our study, we observed a decrease in intracellular SAM (a metabolite of methionine) levels upon knockdown of ZNG1 or METAP1. However, whether the increase in SAM levels is derived from the metabolism of N‐terminal methionine of nascent proteins remains to be further investigated. Furthermore, Hu et al. reported that METAP1 inhibition slows down cell cycle progression, activates the G2/M checkpoint, and induces apoptosis.^[^ ^37^ ^]^ Consistent with these findings, our data indicate that METAP1 deficiency leads to decreased cell viability.

It has been reported that the amino acids histidine, cystine, glutamine, and threonine protect astrocytes from zinc toxicity, with histidine exhibiting the strongest protective effect, followed by cysteine.^[^ ^38^ ^]^ Accordingly, we found that histidine significantly antagonizes the effects of zinc in gut cells. Several studies have reported that histidine, histidine residues (e.g., carnosine), and histidine‐rich proteins have the ability to form complexes with metal ions, including Fe^2+^, Cu^2+^, Co^2+^, Ni^2+^, Cd^2+^, and zinc.^[^ ^39^ ^]^ For example, histidine‐rich glycoproteins bind to zinc and play an important role in immunity,^[^ ^39^, ^40^ ^]^ and carnosine prevents Cu^2+^‐ and zinc‐induced neurotoxicity.^[^ ^41^ ^]^ These studies suggest that histidine has a role in chelating zinc. However, there is no direct evidence in our study that histidine is able to directly chelate Zn and, thus, modulate AKT methylation and activity. This is a limitation of our current findings and a topic that requires further research.

Studies in *C. elegans* have shown mutations in the histidine ammonia‐lyase (haly‐1) gene, which is responsible for converting histidine to ammonia and urocanic acid, as well as higher histidine levels and enhanced zinc tolerance in haly‐1 mutant worms.^[^ ^42^ ^]^ Our findings show that knocking down ZNG1 (a zinc transporter protein) blocks zinc‐mediated activation of AKT. In *Acinetobacter nnii*, the ZNG1 homolog ZigA is considered a zinc chaperone for histidine ammonia‐lyase, and in its absence, histidine levels are known to increase. Thus, the ZNG1‐histidine axis may be involved in regulating zinc concentrations.^[^ ^43^ ^]^ However, whether ZNG1 knockout affects intracellular histidine concentrations, thereby influencing the efficacy of zinc, remains to be further explored.

In summary, our study demonstrates that zinc activates AKT pathway. Mechanically zinc upregulates intracellular SAM levels through the ZNG‐METAP1 axis and leads to PRMT5‐mediated methylation of AKT at sites R15 and R391. Thereby, zinc‐mediated AKT methylation enhances AKT localization and its binding to mTORC2. This mechanism ultimately affects gut cell proliferation and the expression of gut barrier genes and explains the alleviate gut barrier dysfunction effect of zinc.

## 4 Experimental Section

### Antibodies and Reagents

DMEM (amino acid‐free) was purchased from Genetimes Technology (Shanghai, China). Zinc sulfate (ZnSO_4_, Zn (NO_3_)_2_, and ZnCl_2_) was purchased from Bodi (Tianjin, China). Fetal bovine serum (F2442), arginine (A8094), phenylalanine (P5482), methionine (M2768), threonine (T8441), histidine (H6034), leucine (L8912), valine (V0500), and lysine (L8662) were obtained from Sigma‐Aldrich (MO, USA).

Antibodies against p‐S6 (4858S), S6 (2217S), pS473‐AKT (9271), pT308‐AKT (4056), AKT (9272), p‐GSK3β (5558), GSK3β (9315) and PRMT5(79 998) were obtained from Cell Signaling Technology (MA, USA). Anti‐Flag Tag (db7002) and Anti‐HA Tag (db2603) were obtained from Diagbio (Hangzhou, China). Pan‐Symmetric Di‐Methyl Arginine Motif Rabbit mAb (A20794) and Anti‐Ki67 (ab15580) were obtained from ABclonal (Wuhan, China). ZO‐1 (21773‐1‐AP), occludin (27260‐1‐AP), claudin1 (28674‐1‐AP), METAP1 (28702‐1‐AP), GPR39 (23326‐1‐AP) and E‐cadherin (20874‐1‐AP) were purchased from Proteintech (Wuhan, China). ZIP1 (PA5‐21066) was purchased from Thermo Fisher Scientific (MA, USA). Amino acids (50×) was purchased from Gibco (Grand Island, NY, USA). Phosphate‐buffered saline (PBS) and trypsin were purchased from HyClone (UT, USA). Cell Counting Kit‐8 (CCK‐8) (K009) was purchased from ZETA LIFE (CA, USA). TPEN (HY‐100202), GSK3326595 (HY‐101563) and SCH772984 (HY‐50846) were obtained from MedChemExpress (NJ, USA).

### Cell Culture

The IPEC‐J2 (RRID: CVCL_WG02) cell line, an intestinal epithelial cell line, was kindly donated by South China Agricultural University. The colorectal cancer cell lines RKO (RRID: CVCL_0504), SW620 (RRID: CVCL_0547), along with the hepatocellular carcinoma cell line HepG2 (RRID: CVCL_0027), were generously shared by Tongji University. The non‐small cell lung cancer cell line H1299 (RRID: CVCL_0060), the colorectal carcinoma cell line HCT116 (RRID: CVCL_0291), and the human embryonic kidney cell line 293T HEK293T (RRID: CVCL_0063) were all purchased from the National Basic Science Data Center for Basic Sciences in China. The cell culture process was strictly carried out according to the instructions in the manual. All cell lines were cultured in Dulbecco's Modified Eagle Medium containing 10% fetal bovine serum that was maintained at a constant temperature of 37 °C and a 5% carbon dioxide environment.

### Plasmid Construction

Flag‐AKT and Flag‐SIN1 were generated by cloning the cDNA sequence of the target gene into the pcDNA3.1‐Flag vector. HA‐AKT was produced by subcloning the target DNA fragment into the HA vector. The Flag‐AKT‐R15K/R391K recombinant plasmid was constructed using PCR site‐directed mutagenesis.

### siRNA Knockdown

Both non‐specific control siRNA and specific siRNAs were purchased from GenePharma (Shanghai, China). Lipofectamine 2000 (11 668 030, Thermo Fisher Scientifi, MA, USA) and siRNA were transfected into cells at a 1:1 ratio. The siRNA sequences were as follows.

METAP1: 5ʹ‐CTCACAAGTTACTACATAAGAAA‐3ʹ
PRMT5: 5ʹ‐CTGAATTGCGTCCCCGAAATAGC‐3ʹ
ZNG1: 5ʹ‐CCCAGCGGTTCAGCTGAGGTAGG‐3ʹ
ZIP1: 5ʹ‐CTGCCAATTTTGGTATCTTCTCT‐3ʹ
GPR39: 5ʹ‐GTGCGGGCTACCTTACTGAAC‐3ʹ

### Western Blot Analysis

For cell samples, a certain amount of loading lysis buffer was added to the mixture and left to stand at room temperature for 5 min before being collected into corresponding EP tubes. Frozen gut tissue was homogenized and disrupted in pre‐cooled RIPA lysis buffer, and this was followed by centrifugation at 12,000 rpm for 15 min. The supernatant was collected for western blot analysis. The total protein concentration was determined using a BCA protein assay kit (TIANGEN, Beijing, China).

The collected protein samples were boiled at 100 °C for 15 min, separated by polyacrylamide gel electrophoresis, and transferred onto a 0.45 µm nitrocellulose membrane using the sandwich transfer method. The membrane was then blocked with 5% skim milk at room temperature for 1 h. It was next incubated overnight with the primary antibody and then the corresponding secondary antibody. Finally, the protein bands were detected under an imaging system using an ECL luminescent solution, and the protein grayscale was quantified using the ImageJ software.

### Co‐Immunoprecipitation Analysis

After 24 h of transfection with specific plasmids, the cells were lysed using ice‐cold lysis buffer containing phosphatase inhibitors (Na₃VO₄ and NaF) and protease inhibitors. The cell lysate was then centrifuged, and the supernatant was incubated with anti‐Flag M2 magnetic beads for 4–6 h. Subsequently, the immunoprecipitate was washed three times with lysis buffer and then analyzed by immunoblotting with the designated antibodies after addition of the loading buffer.

### qRT‐PCR Analysis

The tissues were first homogenized, and then RNA was extracted from the tissue homogenates and cells using TRIzol reagent (Takara, Dalian, China). Next, the RNA was separated and purified according to the manufacturer's instructions. Subsequently, the extracted RNA was efficiently reverse‐transcribed into cDNA using the PrimerScript RT kit (RR047A; Takara, Dalian, China).

For qRT‐PCR analysis, the TB Green qRT‐PCR kit (RR820A, Takara, Dalian, China) and the Roche LightCycler®96 qRT‐PCR System (Roche, Germany) were employed to ensure the precision of the experimental results. Three technical replicates were performed for each sample, and each sample was run twice to minimize errors and guarantee the accuracy of the results. Using the Ct (cycle threshold) method and β‐actin as the internal reference gene, the relative expression levels of mRNA was accurately calculated. The cycling conditions for PCR amplification were as follows: initial denaturation at 95 °C for 30 s followed by 40 cycles each of denaturation at 95 °C for 10 s and annealing/extension at 60 °C for 30 s. These conditions were carefully determined based on the specificity of the primers and the needs of the experiment, with the aim of ensuring the specificity and efficiency of the PCR reaction. The specific sequences of the PCR primers are detailed in Table S1 (Supporting Information). They were carefully designed based on the sequences of the target genes.

### Cell Viability Assay

Cell viability was assessed using the CCK‐8 kit. Cells were seeded at a concentration of 5 × 10^3^ cells per well in a 96‐well plate and treated with varying concentrations of zinc. Subsequently, the CCK‐8 reagent was added to the wells, and the cells were incubated for 2–3 h at 37 °C. Thereafter, the optical density values were measured using a Multifunctional enzyme label instrument (BioTek, USA) at a wavelength of 450 nm to evaluate cell viability.

### Animals and Experimental Design

All experimental procedures involving piglets and mice in this study were approved by the Institution Animal Care and Use Committee of the Northwest A&F University (approval no. NWAFU‐2020‐1131).

### Animals and Experimental Design—The Piglet Experiments

The piglet experiments were conducted at the Animal Husbandry Teaching and Research Base of Northwest A&F University. In Experiment 1, 16 weaned piglets (Duroc × Landrace × Large White), eight males and eight females that were 25 days old and weighed ≈8.53 kg, were randomly divided into two treatment groups: the control group (basic diet) and the ZnO group (basic diet with the addition of 2500 mg kg^−1^ ZnO). Each treatment was performed in eight replicates, with one piglet per replicate, and the piglets were housed in separate pens within the same barn under similar conditions. The day on which feeding commenced was marked as day 0 of the experiment. On day 6, four piglets were selected for slaughter and sampling. During the experiment, the initial weight of the pigs was recorded, and feeding was done at fixed times and at fixed quantities. The health status of the piglets was regularly observed through daily records of fecal conditions in each pen.

In Experiment 5, another set of 16 weaned piglets (Duroc × Landrace × Large White) of similar age and weight (half male and half female) were randomly assigned to two treatments groups: the high‐protein diet group (HDP) and the low‐protein diet group (LDP). The experimental setup and feeding conditions were the same as those in Experiment 1. After the piglets were slaughtered, tissues from the duodenum, jejunum, ileum, and colon were quickly collected and washed with PBS. Some of the tissues were fixed in a 4% paraformaldehyde solution for subsequent preparation for hematoxylin and eosin (H&E) staining. The remaining tissues were placed in cryovials, and the cryovials were tightly sealed and then rapidly immersed in liquid nitrogen. Finally, they were transferred to a ‐80 °C freezer for storage.

### Animals and Experimental Design—The Mice Experiments

In Experiment 2, 18 C57BL/6 mice with comparable body weights were randomly allocated into three groups, each comprising six animals. All mice were maintained on a zinc‐deficient diet and provided with 2% dextran sodium sulfate (DSS) in drinking water to induce gut barrier dysfunction throughout the experiment. Group 1 (DSS group) received daily oral gavage of 0.2 mL sterile phosphate‐buffered saline (PBS). Meanwhile, Group 2 (DSS+ZnO NPs (5 mg kg^−1^)) and Group 3 (DSS+ZnO NPs (10 mg kg^−1^)) were administered ZnO nanoparticle solutions at doses of 5 and 10 mg kg^−1^ body weight, respectively, diluted in PBS and delivered via oral gavage based on their individual body weights each day. The experimental duration was six days, after which the mice were anesthetized and euthanized by cervical dislocation on the seventh day. Colon tissues were promptly excised and rinsed with PBS; some samples were fixed in 4% paraformaldehyde for histological processing and preparation of H&E‐stained sections. Remaining tissue samples were placed in cryovials, sealed tightly, and immediately submerged in liquid nitrogen before being transferred to a ‐80 °C freezer for long‐term storage and subsequent analysis.

In Experiment 3, 18 C57BL/6 mice with comparable body weights were randomly assigned to three groups, each consisting of six animals. All mice were maintained on a histidine‐deficient diet and provided with 2% DSS in drinking water. Each mouse received a daily gavage of 10 mg ZnO NPs per kilogram of body weight. Group 1 (DSS group) continued to receive DSS water as provided. Meanwhile, Group 2 (His‐0.5) and Group 3 (His‐1.0) had their drinking water supplemented with histidine at concentrations of 0.5 and 1.0 g L^−1^, respectively, to investigate the potential antagonistic effects of histidine on ZnO NPs. Colon tissue samples were processed according to previously described methods.

In Experiment 4, 24 C57BL/6 mice with comparable body weights were randomly assigned to four groups for a 2 × 2 factorial design experiment. All mice were fed a diet deficient in both histidine and zinc and provided with 2% DSS in drinking water throughout the experiment to induce gut barrier dysfunction.

The levels of zinc supplementation were defined as low (5 mg kg^−1^) and high (10 mg kg^−1^), while the levels of histidine supplementation in the drinking water were set at low (0.5 g L^−1^) and high (1.0 g L^−1^). The four groups in the 2 × 2 factorial designs were as follows: Group 1: Low Histidine (L‐His) + Low Zinc (L‐Zinc); Group 2: Low Histidine (L‐His) + High Zinc (H‐Zinc); Group 3: High Histidine (H‐His) + Low Zinc (L‐Zinc); Group 4: High Histidine (H‐His) + High Zinc (H‐Zinc). Colon tissue samples were processed according to previously described methods.

In Experiment 5, thirty C57BL/6 mice were randomly divided into three groups (n = 10 per group) and all received drinking water containing 2% DSS throughout the experiment to induce colitis. The control group (n = 6) was administered 0.2 mL of PBS by oral gavage daily. The DSS + ZnO NPs group received daily oral gavage of zinc oxide nanoparticles (ZnO NPs, 10 mg kg^−1^) for six consecutive days. The ZnO NPs + MK‐2206 group was additionally treated with intraperitoneal injections of the AKT inhibitor MK‐2206 (120 mg kg^−1^) on days 2 and 4.

### Assessment of Diarrhea in Piglets

Piglets were regularly observed at 9:00 AM and 4:00 PM during the experiment to monitor diarrhea conditions using a fecal scoring system. The scoring system utilized a 5‐point scale, with scores ranging from 1 (for granular and hard feces) to 5 (for watery or mucus‐like feces). Diarrhea in piglets was defined as a fecal score of 4–5 for two consecutive days. The incidence of diarrhea was calculated using the following formula: number of diarrhea occurrences among piglets / (the total number of piglets in the experiment × the total number of experimental days) × 100%.

### Immunohistochemistry

Colon tissue samples were fixed in 4% paraformaldehyde for 24 h to ensure proper fixation. After fixation, the tissue samples were dehydrated, cleared, and embedded with paraffin wax. The tissues were then sectioned, mounted on slides, dewaxed, and rehydrated. This was followed by antigen retrieval, blocking of nonspecific binding, primary antibody incubation, washing, and secondary antibody/marker incubation. Finally, a color reaction was performed, and the tissue sections were sealed for observation of morphological and fluorescence imaging under an optical microscope.

### H&E Staining

The colon tissue was fixed in a 4% paraformaldehyde solution for 24 h and subsequently rinsed with running water. Following the rinsing process, the tissues were dehydrated through an ascending series of alcohol concentrations: 50%, 60%, 70%, 90%, 95%, and finally 100% anhydrous ethanol. The dehydrated tissues were then immersed in a xylene solution to achieve tissue transparency. The transparent tissues were embedded in liquid paraffin, dewaxed, sliced, dyed, and sealed. Finally, images of different fields of view were captured at different magnifications using an inverted fluorescence microscope (Olympus, Japan).

### Measurement of SAM

The SAM content in zinc‐stimulated cells was quantified using a SAM ELISA Kit (MM‐61728H2, Meimian Biotechnology, Jiangsu, China). The cell culture supernatant, negative control, and standard products at different dilutions were added to a 96‐well plate coated with antibodies for incubation according to the manufacturer's instructions. The subsequent steps involved washing of the plate, addition of enzyme‐labeled antibodies, substrate color development, termination of the reaction, and determination of the readings.

### Cytoplasmic Membrane Separation

After cell lysis, proteins were isolated from different cell compartments using the Subcellular Protein Fractionation Kit (78 840, Thermo Fisher). Initially, the cytoplasmic components were isolated through gentle centrifugation at 4 °C. Then, the precipitates were treated with pre‐cooled membrane extraction buffer (MEB) to extract membrane proteins via vortex mixing, incubation, and centrifugation. Subsequently, the residual precipitate was then incubated with cold nuclear extraction buffer (NEB) containing protease inhibitors at 4 °C for 30 min under gentle agitation to extract soluble nuclear proteins, before being centrifuged at 5000 × g for 5 min. Finally, the chromatin‐binding buffer, combined with NEB, protease inhibitors, CaCl₂, and micrococcal nuclease at room temperature, was used to extract chromatin‐bound proteins after a 15‐min incubation and 5‐min centrifugation at 16000 × g. Next, the leftover precipitate, upon mixing with Pellet Extraction Buffer (PEB), at room temperature and subsequent 10‐min incubation, yielded cytoskeleton extracts following a 5‐min centrifugation at 16000 × g, with the supernatant being collected into a new tube.

### Amino Acid Determination

The cell samples were homogenized, and 500 µL of water and 500 µL of methanol were added to the homogenized solution. Vigorous oscillation and extraction were performed for 0.5 h, and this was followed by centrifugation at 4 °C for 5 min at 12 000 r min^−1^. Then, 20 µL of the supernatant was transferred into a labeled 1.5‐mL centrifuge tube, to which 40 µL of isopropyl alcohol (1% formic acid) was added and mixed for 60 min. After another round of centrifugation at 4 °C and 12 000 r min^−1^ for 5 min, the supernatant was collected and analyzed using Liquid Chromatography‐Quadrupole Ion Trap‐Mass Spectrometry (LC‐QTRAP‐MS, AB SCIEX, MA, USA).

### Transcriptome Sequencing

Transcriptome sequencing was done by Lianchuan Biotechnology Co. Ltd. (Hangzhou, China). The data produced by sequencing were called raw reads. Next, a quality control analysis of the original read length was performed to assess the quality and suitability of the sequencing data for subsequent bioinformatics analysis. After quality control, the hierarchical indexing (HISAT2) method was used for transcript splicing and alignment to accurately compare the filtered high‐quality read segments with the reference genome. For the analysis of differentially expressed genes (DEGs), the limma package was used for statistical modeling and made appropriate adjustments to the results. Specifically, genes with | log2‐fold variation | greater than 1 and an adjusted P‐value less than 0.05 were defined as DEGs.

### Detection of Zinc Chelating

Zinc ion chelating power of histidine was measured by a UV‐1750 spectrophotometer (Shimadzu, Japan) with using 1 cm path length quartz cuvette at room temperature. Zn^2+^ stock solution was prepared in double distilled water. The histidine alone or in the presence of ZnSO_4_ were incubated at room temperature, and absorbance values were performed in the 200–500 nm wavelength range.

### Measurement of Intracellular Zinc

HEK293T cells were starved in amino acid‐free medium for 1 h and then supplemented with 50 µm ZnSO_4_ for 1 h, either alone or combined with histidine After stimulation, the transfected cells were incubated with 5 µm FluoZin‐3 AM (F24195, Thermo Fisher Scientifi, MA, USA), a cell‐permeable zinc fluorophore, in the medium without FBS for 30 min, and intracellular Zn^2+^ accumulation was measured by fluorescence microscopy as described above.

### Determination of Total Zinc in Tissues

The total zinc content in the tissues was determined using the Zinc (Zn) Colorimetric Assay Kit (E‐BC‐K137‐M, Elabscience, Wuhan, China). An appropriate amount of fresh animal tissue block was taken, rinsed with PBS to remove the blood, wiped dry with filter paper, weighed, and put into the Homogenization container, according to the ratio of animal tissue weight (g): PBS (mL) = 1:9, low‐temperature homogenization. 4 °C, 10,000 rpm centrifugation for 10 min, take the supernatant on ice to be tested. According to the test instructions, add the color development solution for colorimetry, and finally quantified according to the standard curve.

### Metabolome Analysis

Meiwei Metabolism Co. Ltd. (Wuhan, China) performed non‐targeted metabolomics analyses. Liquid chromatographic separation was achieved using an ultra‐high performance liquid chromatography system from Shimadzu (Japan), and mass spectrophotometry analysis was performed on a high‐resolution mass spectrometer (timsTOF Pro; Foster City, CA, USA). Chromatography was performed with a Waters ACQUITY UPLC BEH C18 column (1.8 µm pore size, 2.1 mm × 100 mm) with ultra‐pure water containing 0.1% formic acid as mobile phase A and acetonitrile containing 0.1% formic acid as mobile phase B. The column temperature was set at 40 °C, and the flow rate and sample volume were 0.40 mL min^−1^ and 2 µL, respectively. After pretreatment, the metabolites identified were analyzed using the laboratory's self‐built database, public resource databases, artificial intelligence prediction databases, and the mtDNA method.

### Immunofluorescence (IF) Staining

Cells cultured on glass coverslips were fixed with pre‐chilled methanol at –20  °C in the dark for 30 min. Following fixation, residual aldehydes were quenched using 0.1 M ammonium chloride (NH_4_Cl) for 10 min at room temperature. Cells were then permeabilized with 0.5% Tween‐20 in PBS for 15 min, and subsequently blocked with 1% bovine serum albumin (BSA) for 1 h at room temperature. Primary antibodies (anti‐AKT, anti–E‐Cadherin) were incubated overnight at 4 °C, followed by incubation with fluorophore‐conjugated secondary antibodies for 1 h at room temperature in the dark. Nuclei were counterstained with DAPI (1 µg mL^−1^) for 5 min. Coverslips were mounted with antifade medium and visualized using a Laser Scanning Confocal Microscopy (GERMANY, LEICA, LEICA TCS SP8).

### Isothermal Titration Calorimetry (ITC)

The binding interaction between ZnSO_4_ and histidine was analyzed using a Nano‐ITC calorimeter (Waters Corporation, USA). All solutions were degassed prior to titration. Histidine (1 mm) was loaded into the syringe and titrated into a sample cell containing 100 µm ZnSO_4_ in the same buffer. A series of injections (typically 19 × 2 µL) were performed at 25 °C with stirring at 750 rpm. The resulting thermograms were analyzed using the MicroCal Origin software, and binding parameters, including the dissociation constant (K_d) and stoichiometry (n), were obtained by fitting the data to a one‐site binding model.

### Enzyme‐Linked Immunosorbent Assay (ELISA)

Colon tissues were homogenized in cold PBS containing protease inhibitors and centrifuged at 12000 × g for 10 min at 4 °C. The supernatants were collected, and the concentrations of inflammatory cytokines were measured using commercially available ELISA kits (Fankewell, Shanghai, China), according to the manufacturer's instructions. Absorbance was read at 450 nm using a multifunctional microplate reader (BioTek, USA).

### Statistical Analysis

GraphPad Prism 8.0 was used for all the data analyses. Data were presented as the mean ± SD. The statistical tests included unpaired one‐tailed or two‐tailed Student *t*‐test and one‐way analysis of variance. A p‐value of 0.05 was considered to indicate statistical significance.

## Conflict of Interest

The authors declare no conflict of interest.

## Author Contributions

C.C., Y.Z., B.S., G.W., L.D., and S.Q. performed conceptualization. C.C., Y.Z., B.S., G.W., P.L., H.G., R.L., M.Z., Y.Z., and D.F. performed methodology. C.C., Y.Z., B.S., G.W., and L.D. performed wrote the original draft. C.C., Y.Z., B.S., G.W., P.L., H.G., R.L., M.Z., Y.Z., and D.F. performed investigation. L.C., G.C., L.D., and S.Q. performed supervision, project administration, Funding acquisition, and provided resources. L.C., G.C., L.D., and S.Q. performed wrote, reviewed, edited the draft.

## Supporting information



Supporting Information

## Data Availability

The data that support the findings of this study are available in the supplementary material of this article.
