# Supplementary material for: Zinc Alleviates Gut Barrier Dysfunction by Promoting the Methylation of AKT
Source: Adv Sci (Weinh). 2025 Jul 11;12(33):e08280. doi: 10.1002/advs.202508280 (PMC12412578; doi:10.1002/advs.202508280)
Supplement: Supplementary file 1 — Supporting Information [file ADVS-12-e08280-s001.pdf]

ADVANCED SCIENCE
Open Access

Supporting Information

for *Adv. Sci.*, DOI 10.1002/advs.202508280

Zinc Alleviates Gut Barrier Dysfunction by Promoting the Methylation of AKT

Chuanjiang Cai, Yining Zheng, Bo Sun, Guoyan Wang, Pengfei Li, Huijun Geng, Rongnuo Li, Miaomiao Zhu, Yuanyuan Zhu, Dingping Feng, Lei Chen, Guiyan Chu, Lu Deng* and Shiyan Qiao**

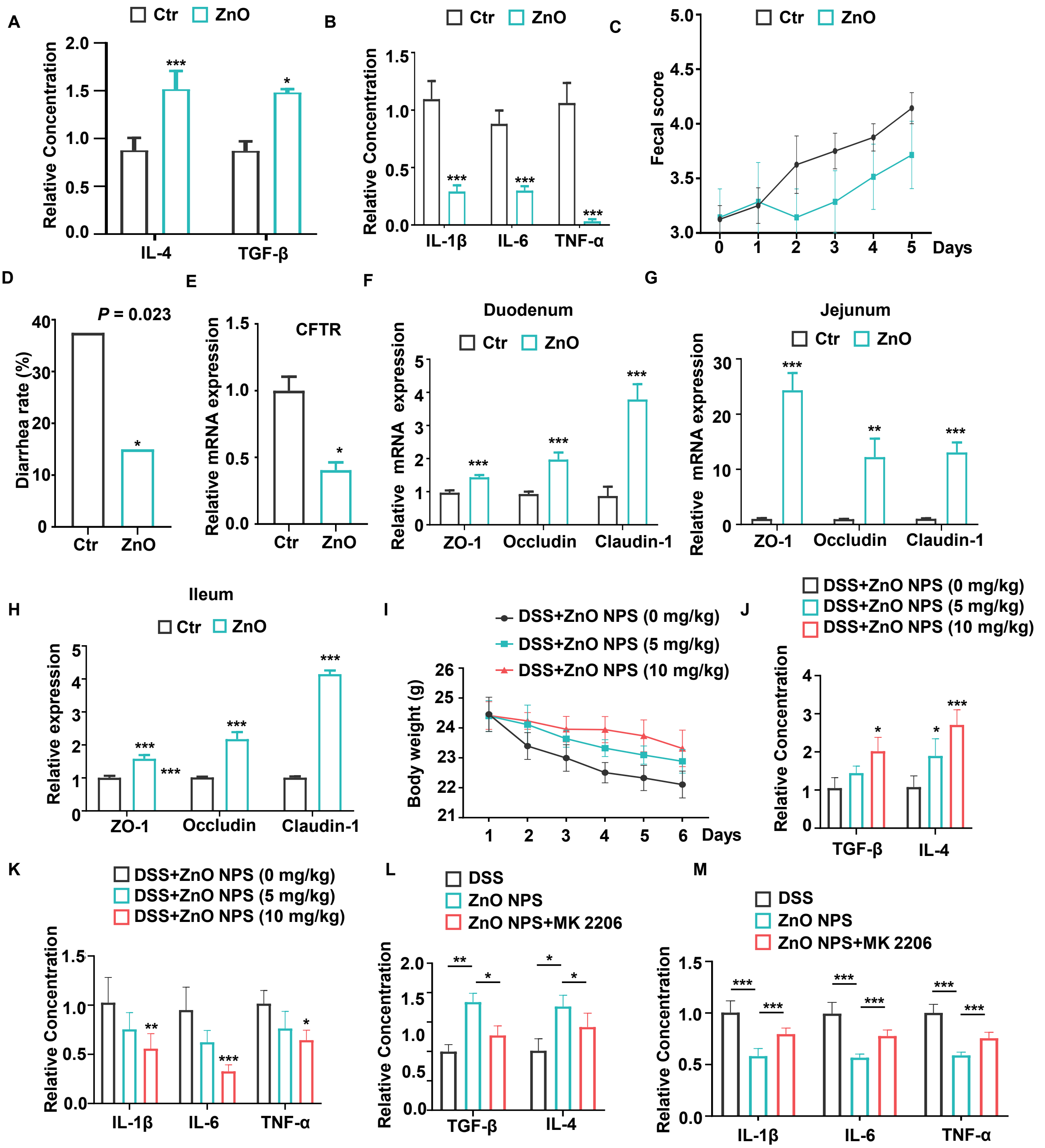
A
Ctr
ZnO
Relative Concentration
2.0
1.5
1.0
0.5
0.0
***
*
IL-4
TGF-β
B
Ctr
ZnO
Relative Concentration
1.5
1.0
0.5
0.0
***
***
***
IL-1β
IL-6
TNF-α
C
Ctr
ZnO
Fecal score
5.0
4.5
4.0
3.5
3.0
0
1
2
3
4
5
Days
D
P = 0.023
Diarrhea rate (%)
40
30
20
10
0
*
Ctr
ZnO
E
CFTR
Relative mRNA expression
1.5
1.0
0.5
0.0
*
Ctr
ZnO
F
Duodenum
Ctr
ZnO
Relative mRNA expression
5
4
3
2
1
0
***
***
***
ZO-1
Occludin
Claudin-1
G
Jejunum
Ctr
ZnO
Relative mRNA expression
30
20
10
0
***
**
***
ZO-1
Occludin
Claudin-1
H
Ileum
Ctr
ZnO
Relative expression
5
4
3
2
1
0
***
***
***
***
ZO-1
Occludin
Claudin-1
I
DSS+ZnO NPS (0 mg/kg)
DSS+ZnO NPS (5 mg/kg)
DSS+ZnO NPS (10 mg/kg)
Body weight (g)
26
25
24
23
22
21
1
2
3
4
5
6
Days
J
DSS+ZnO NPS (0 mg/kg)
DSS+ZnO NPS (5 mg/kg)
DSS+ZnO NPS (10 mg/kg)
Relative Concentration
4
3
2
1
0
*
*
***
TGF-β
IL-4
K
DSS+ZnO NPS (0 mg/kg)
DSS+ZnO NPS (5 mg/kg)
DSS+ZnO NPS (10 mg/kg)
Relative Concentration
1.5
1.0
0.5
0.0
**
***
*
IL-1β
IL-6
TNF-α
L
DSS
ZnO NPS
ZnO NPS+MK 2206
Relative Concentration
2.0
1.5
1.0
0.5
0.0
**
*
*
*
TGF-β
IL-4
M
DSS
ZnO NPS
ZnO NPS+MK 2206
Relative Concentration
1.5
1.0
0.5
0.0
***
***
***
***
***
***
IL-1β
IL-6
TNF-α

Fig. S1 Role of zinc in the alleviation of gut barrier dysfunction and activation of AKT.

(A). The expression levels of IL-4 and TGF-β in the colon tissue of piglet were detected by ELISA. **(B).** The expression levels of IL-1β, IL-6 and TNF-α in the colon tissue of piglet were detected by ELISA. **(C).** Statistics of piglet fecal score from the control group and ZnO treatment group. **(D).** Statistics of piglet diarrhea rate from the control group and ZnO treatment group. **(E).** The expression levels of CFTR in the colon tissue of piglets from the control group and ZnO treatment group were detected by qRT-PCR. **(F-H).** The expression levels of ZO-1, occludin and claudin-1 in the duodenum tissue (F), jejunum tissue (G) and ileum (H) of piglets from the control group and ZnO treatment group were detected by qRT-PCR. **(I).** Body weight of the control group, the DSS + ZnO NPs (5 mg/kg) group and the DSS + ZnO NPs (10 mg/kg) group. **(J).** The expression levels of IL-4 and TGF-β in the colon tissue of mice were detected by ELISA. **(K).** The expression levels of IL-1β, IL-6 and TNF-α in the colon tissue of mice were detected by ELISA. **(L).** The expression levels of IL-4 and TGF-β in the colon tissue of mice were detected by ELISA. **(M).** The expression levels of IL-1β, IL-6 and TNF-α in the colon tissue of mice were detected by ELISA. （*$P<0.05$, **$P<0.01$, ***$P<0.001$）

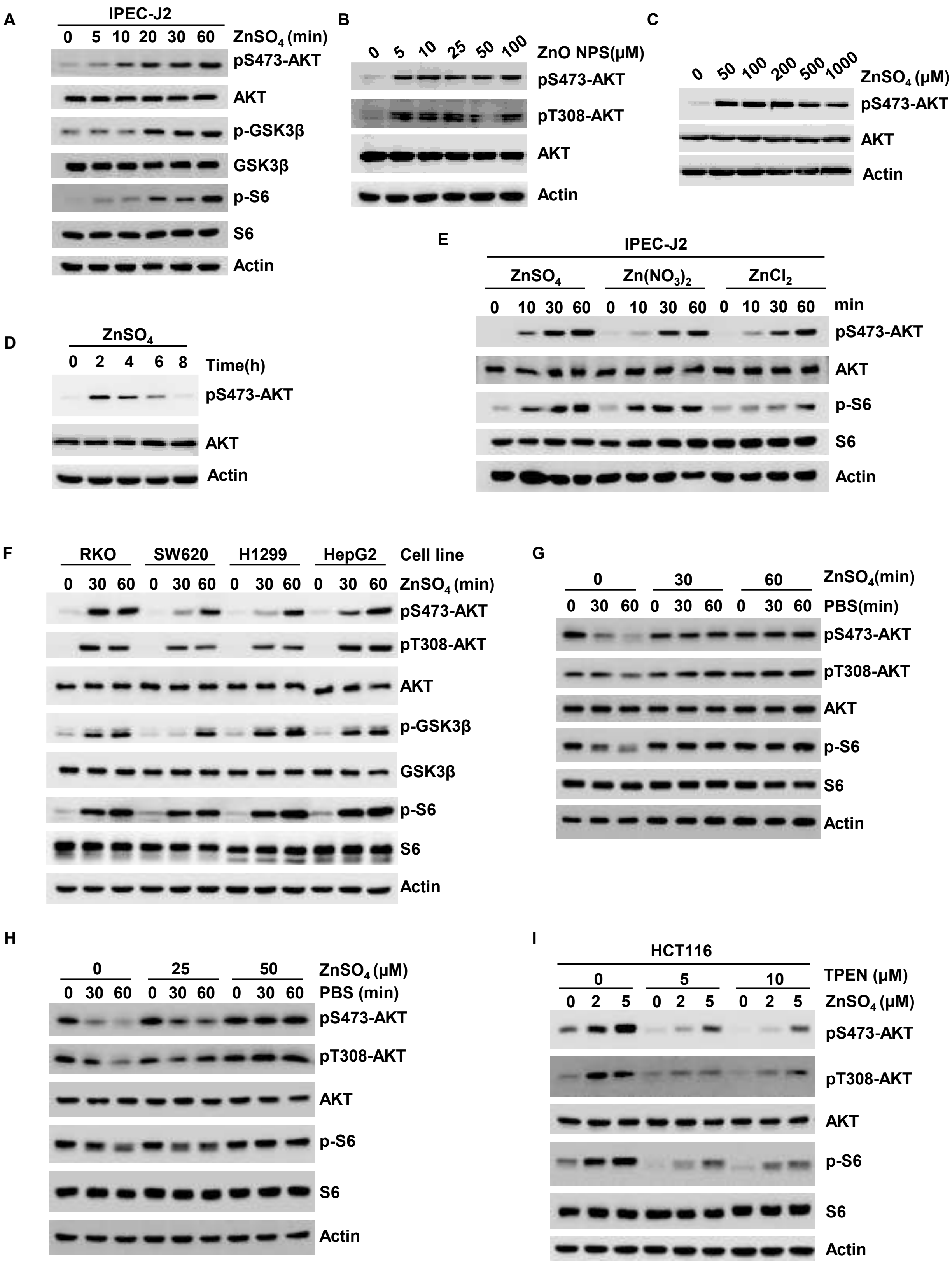
A
IPEC-J2
0 5 10 20 30 60 ZnSO4 (min)
pS473-AKT
AKT
p-GSK3β
GSK3β
p-S6
S6
Actin
B
0 5 10 25 50 100 ZnO NPS(μM)
pS473-AKT
pT308-AKT
AKT
Actin
C
0 50 100 200 500 1000 ZnSO4 (μM)
pS473-AKT
AKT
Actin
D
ZnSO4
0 2 4 6 8 Time(h)
pS473-AKT
AKT
Actin
E
IPEC-J2
ZnSO4 Zn(NO3)2 ZnCl2
0 10 30 60 0 10 30 60 0 10 30 60 min
pS473-AKT
AKT
p-S6
S6
Actin
F
RKO SW620 H1299 HepG2 Cell line
0 30 60 0 30 60 0 30 60 0 30 60 ZnSO4 (min)
pS473-AKT
pT308-AKT
AKT
p-GSK3β
GSK3β
p-S6
S6
Actin
G
0 30 60 ZnSO4(min)
0 30 60 0 30 60 0 30 60 PBS(min)
pS473-AKT
pT308-AKT
AKT
p-S6
S6
Actin
H
0 25 50 ZnSO4 (μM)
0 30 60 0 30 60 0 30 60 PBS (min)
pS473-AKT
pT308-AKT
AKT
p-S6
S6
Actin
I
HCT116
0 5 10 TPEN (μM)
0 2 5 0 2 5 0 2 5 ZnSO4 (μM)
pS473-AKT
pT308-AKT
AKT
p-S6
S6
Actin

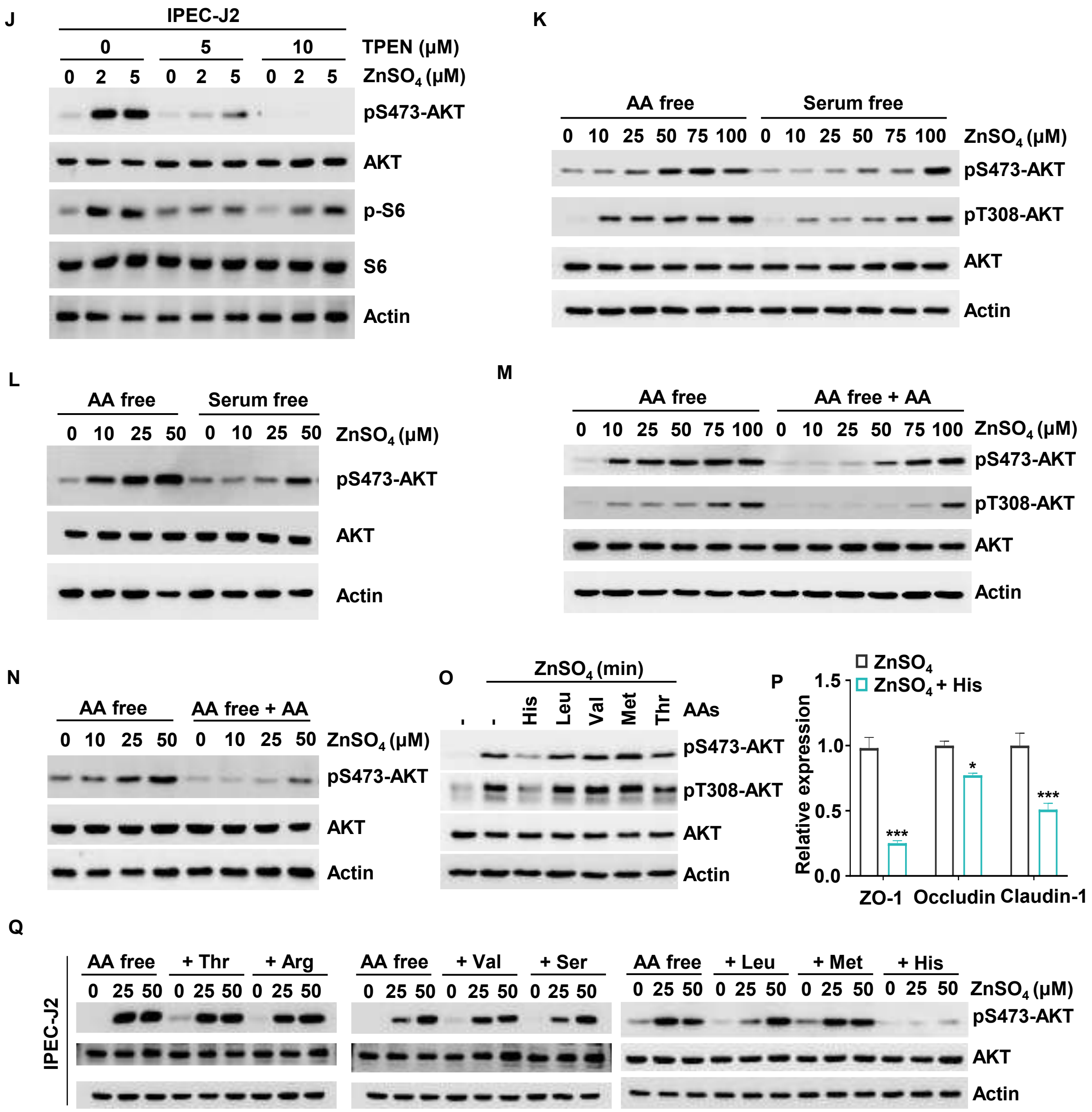
J
IPEC-J2
TPEN (μM)
ZnSO4 (μM)
pS473-AKT
AKT
p-S6
S6
Actin
K
AA free
Serum free
ZnSO4 (μM)
pS473-AKT
pT308-AKT
AKT
Actin
L
AA free
Serum free
ZnSO4 (μM)
pS473-AKT
AKT
Actin
M
AA free
AA free + AA
ZnSO4 (μM)
pS473-AKT
pT308-AKT
AKT
Actin
N
AA free
AA free + AA
ZnSO4 (μM)
pS473-AKT
AKT
Actin
O
ZnSO4 (min)
His
Leu
Val
Met
Thr
AAs
pS473-AKT
pT308-AKT
AKT
Actin
P
ZnSO4
ZnSO4 + His
Relative expression
ZO-1
Occludin
Claudin-1
Q
IPEC-J2
AA free
+ Thr
+ Arg
+ Val
+ Ser
+ Leu
+ Met
+ His
ZnSO4 (μM)
pS473-AKT
AKT
Actin

Fig. S2 Histidine antagonizes zinc-induced AKT activation.

(A). IPEC-J2 cells were starved in PBS for 1 h, and then supplemented with $ZnSO_4$ for the indicated time. The level of pS473-AKT, p-GSK3β, p-S6 and the indicated protein was detected by WB.**(B).** HCT116 cells were starved in PBS for 1 h, and then supplemented with ZnO NPs at the indicated concentration for the 1 h. The level of pS473-AKT, pT308-AKT and the indicated protein was detected by WB. **(C-D).** HCT116 cells were starved in PBS for 1 h, and then supplemented with $ZnSO_4$ at the indicated concentration for the indicated time. The level of pS473-AKT and the indicated protein was detected by WB. **(E).** IPEC-J2 cells were starved of PBS for 1 h and then supplemented with ZnSO4, Zn(NO3)2 or ZnCl2 for the indicated time. The level of pS473-AKT, p-S6 and the indicated protein was detected by WB. **(F).** RKO、SW620、H1299、HepG2 cells were starved in PBS for 1 h , and then supplemented with ZnSO4 for the indicated time. The level of pS473-AKT, pT308-AKT, p-GSK3β, p-S6 and the indicated protein was detected by WB. **(G).** HCT116 cells were starved in PBS for indicate time, and then supplemented with $ZnSO_4$ for the indicated time. The level of pS473-AKT, pT308-AKT, p-S6 and the indicated protein was detected by WB. **(H).** HCT116 cells were starved in PBS for indicate time, and then supplemented with $ZnSO_4$ for the indicated time. The level of pS473-AKT, pT308-AKT, p-S6 and the indicated protein was detected by WB. **(I-J).** HCT116 cells (F) or IPEC-J2 cells (G) were treated with TPEN (0, 5, 10 μM) for 1 h, starved in PBS for 1 h, and then supplemented with $ZnSO_4$ at the indicated concentration. The level of pS473-AKT, pT308-AKT, p-S6 and the indicated protein was detected by WB. **(K).** HCT116 cells were starved in serum-free or amino acid-free medium for 1 h, and then supplemented with $ZnSO_4$ in concentration gradients of 0, 10, 25, 50, 75, and 100 μM for 1 h. The level of pS473-AKT, pT308-AKT and the indicated protein was detected by WB. **(L).** IPEC-J2 cells were starved in serum-free or amino acid-free medium for 1 h, and then supplemented with $ZnSO_4$ at the indicated concentration for 1 h. The level of pS473-AKT and the indicated protein was detected by WB. **(M).** HCT116 cells were starved in amino acid-free medium for 1 h, then supplemented with $ZnSO_4$ in concentration gradients of 0, 10, 25, 50, 75, and 100 μM for 1 h, either alone or combined with amino acids. The level of pS473-AKT, pT308-AKT and indicated protein was analyzed by WB. **(N).** IPEC-J2 cells was starved in amino acid-free medium for 1 h, then supplemented with $ZnSO_4$ at the indicated concentration, either alone or combined with amino acids for 1 h. The level of pS473-AKT and indicated protein was analyzed via WB. **(O).** HCT116 cells were starved in amino acid-free medium, then supplemented with 50 μM $ZnSO_4$, either alone or combined with histidine, leucine, valine, methionine, or threonine for 1 h. The level of pS473-AKT, pT308-AKT and indicated protein was analyzed by WB. **(P).** HCT116 cells were starved in amino acid free medium for 1 h, then supplemented with 50 μM $ZnSO_4$ for 1 h, either alone or combined with histidine. The level of ZO-1, occludin1, claudin1 and indicated protein was analyzed by qRT-PCR. **(Q).** IPEC-J2 cells were starved in amino acid-free medium, then supplemented with $ZnSO_4$ in concentration gradients of 0, 25, and 50 μM, either alone or combined with threonine, arginine, valine, histidine, serine, leucine, methionine, or histidine for 1 h. The level of pS473-AKT, pT308-AKT and indicated protein was analyzed by WB.（$^{*}P<0.05$, $^{**}P<0.01$, $^{***}P<0.001$）

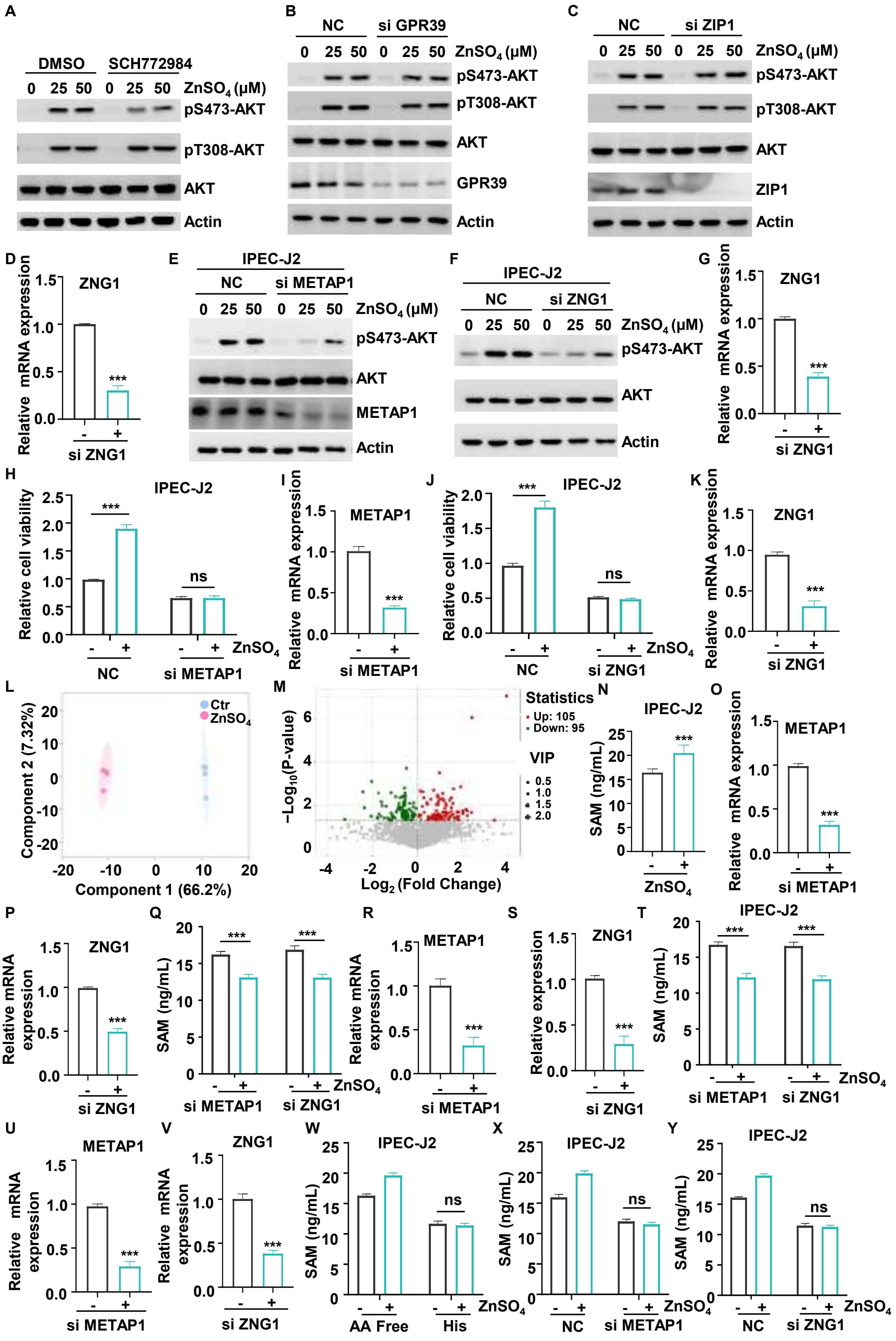
A
DMSO
SCH772984
0 25 50 0 25 50 ZnSO4 (μM)
pS473-AKT
pT308-AKT
AKT
Actin
B
NC
si GPR39
0 25 50 0 25 50 ZnSO4 (μM)
pS473-AKT
pT308-AKT
AKT
GPR39
Actin
C
NC
si ZIP1
0 25 50 0 25 50 ZnSO4 (μM)
pS473-AKT
pT308-AKT
AKT
ZIP1
Actin
D
ZNG1
Relative mRNA expression
***
si ZNG1
E
IPEC-J2
NC
si METAP1
0 25 50 0 25 50 ZnSO4 (μM)
pS473-AKT
AKT
METAP1
Actin
F
IPEC-J2
NC
si ZNG1
0 25 50 0 25 50 ZnSO4 (μM)
pS473-AKT
AKT
Actin
G
ZNG1
Relative mRNA expression
***
si ZNG1
H
IPEC-J2
Relative cell viability
***
ns
ZnSO4
NC
si METAP1
I
METAP1
Relative mRNA expression
***
si METAP1
J
IPEC-J2
Relative cell viability
***
ns
ZnSO4
NC
si ZNG1
K
ZNG1
Relative mRNA expression
***
si ZNG1
L
Ctr
ZnSO4
Component 2 (7.32%)
Component 1 (66.2%)
M
Statistics
Up: 105
Down: 95
VIP
-Log10(P-value)
Log2 (Fold Change)
N
IPEC-J2
SAM (ng/mL)
***
ZnSO4
O
METAP1
Relative mRNA expression
***
si METAP1
P
ZNG1
Relative mRNA expression
***
si ZNG1
Q
SAM (ng/mL)
***
***
ZnSO4
si METAP1
si ZNG1
R
METAP1
Relative mRNA expression
***
si METAP1
S
ZNG1
Relative expression
***
si ZNG1
T
IPEC-J2
SAM (ng/mL)
***
***
ZnSO4
si METAP1
si ZNG1
U
METAP1
Relative mRNA expression
***
si METAP1
V
ZNG1
Relative mRNA expression
***
si ZNG1
W
IPEC-J2
SAM (ng/mL)
ns
ZnSO4
AA Free
His
X
IPEC-J2
SAM (ng/mL)
ns
ZnSO4
NC
si METAP1
Y
IPEC-J2
SAM (ng/mL)
ns
ZnSO4
NC
si ZNG1

Fig. S3 Promotion of AKT activation by zinc through the ZNG1-METAP1 axis. (A). HCT116 cells were treated with SCH772984 for 24 h, starved in PBS for 1 h, and then supplemented with $ZnSO_4$ for the indicated concentration for 1 h. The level of pS473-AKT, pT308-AKT and the indicated protein was detected by WB. **(B-C).** GPR39 (B) and ZIP1A (C) WT or knockdown HCT116 cells were starved in amino acid-free medium and then supplemented with $ZnSO_4$ at the indicated concentration for 1 h. The level of pS473-AKT, pT308-AKT, and the indicated protein was detected by WB. **(D).** The knockdown efficiency of ZNG1 was shown. **(E-G).** METAP1 (E) and ZNG1 (F) WT or knockdown IPEC-J2 cells were starved in amino acid-free medium and then supplemented with $ZnSO_4$ at the indicated concentration for 1 h. The level of pS473-AKT and the indicated protein was detected by WB. The knockdown efficiency of ZNG1 (G) was shown. **(H-K).** METAP1 (H) and ZNG1 (J) WT or knockdown IPEC-J2 cells were treated with of 50 μM $ZnSO_4$ for 48 h. Cell viability was detected using the CCK-8 assay, n = 3. The knockdown efficiency of METAP1 (I) and ZNG1 (K) was shown. **(L).** Principal Component Analysis (PCA) between HPD group and LPD group. **(M).** Volcanic maps of different metabolites between HPD group and LPD group. **(N).** IPEC-J2 cells were starved in amino acid-free medium and then supplemented with 50 μM $ZnSO_4$ for 1 h. The intracellular SAM levels were assayed by Elisa. **(O-Q).** METAP1 and ZNG1 WT or knockdown HCT116 cells were starved in amino acid-free medium and then supplemented with 50 μM $ZnSO_4$ for 1 h. The intracellular SAM levels were assayed by Elisa. The knockdown efficiency of METAP1 (O) and ZNG1 (P) was shown. **(R-T).** METAP1 and ZNG1 WT or knockdown HCT116 cells were starved in amino acid-free medium and then supplemented with 50 μM $ZnSO_4$ for 1 h. The intracellular SAM levels were assayed by Elisa. The knockdown efficiency of METAP1 (R) and ZNG1 (S) was shown. **(U-V).** The knockdown efficiency of METAP1 (U) and ZNG1 (V) was shown. **(W).** IPEC-J2 cells were starved in amino acid-free medium for 1 h, then supplemented with 50 μM $ZnSO_4$ for 1 h, either alone or combined with histidine. The intracellular SAM levels were detected by Elisa. **(X-Y).** METAP1 (X) and ZNG1 (Y) WT or knockdown IPEC-J2 cells were starved in amino acid-free medium and then supplemented with 50 μM $ZnSO_4$ for 1 h. The intracellular SAM levels were detected by Elisa. （*P<0.05, **P<0.01, ***P<0.001）

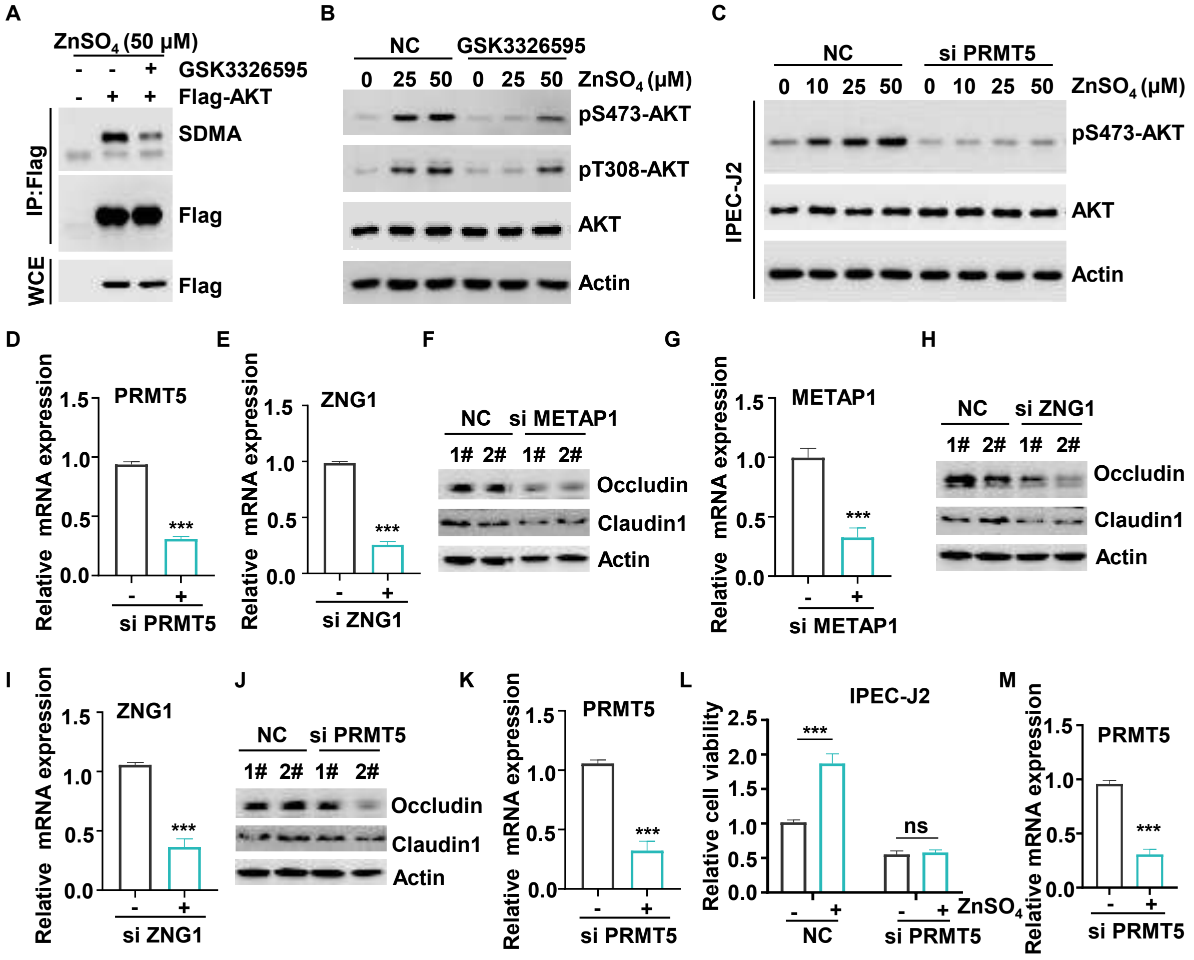

Fig. S4 Protective effect of zinc on the intestinal barrier through PRMT5-mediated methylation of AKT. (A). HEK293T cells were transfected with Flag-AKT. After transfection for 24 h, cells were starved in amino acid-free medium for 1 h, then supplemented with 50 μM $ZnSO_4$ for 1 h, either alone or combined with GSK3326595. AKT was immunoprecipitated and subsequently analyzed by WB for SDMA modification. **(B).** IPEC-J2 cells were treated with GSK3326595 (10 μM) for 6 h, cells were starved in amino acid-free medium for 1 h, and then supplemented with $ZnSO_4$ at the indicated concentration for 1 h. The level of pS473-AKT and indicated protein was analyzed by WB. **(C).** PRMT5 WT or knockdown IPEC-J2 cells were starved in amino acid-free medium for 1 h and then supplemented with $ZnSO_4$ at the indicated concentration for 1 h. The level of pS473-AKT and indicated protein was analyzed by WB. **(D-E).** The knockdown efficiency of PRMT5 (D) and ZNG1 (E) was shown. **(F-K).** METAP1 (F), ZNG1 (H), and PRMT5 (J) WT or knockdown HCT116 cells were starved in amino acid-free medium for 1 h and then supplemented with 50 μM $ZnSO_4$ for 1 h. The expression levels of ZO-1, occludin, and claudin-1 were detected by WB. The knockdown efficiency of METAP1 (G), ZNG1 (I) and PRMT5 (K) was shown. **(L-M).** PRMT5 WT or knockdown IPEC-J2 cells were treated with of 50 μM ZnSO4 for 48 h. Cell viability was detected using the CCK-8 assay, n = 3. The knockdown efficiency of PRMT5 (M) was shown. （*P<0.05, **P<0.01, ***P<0.001）

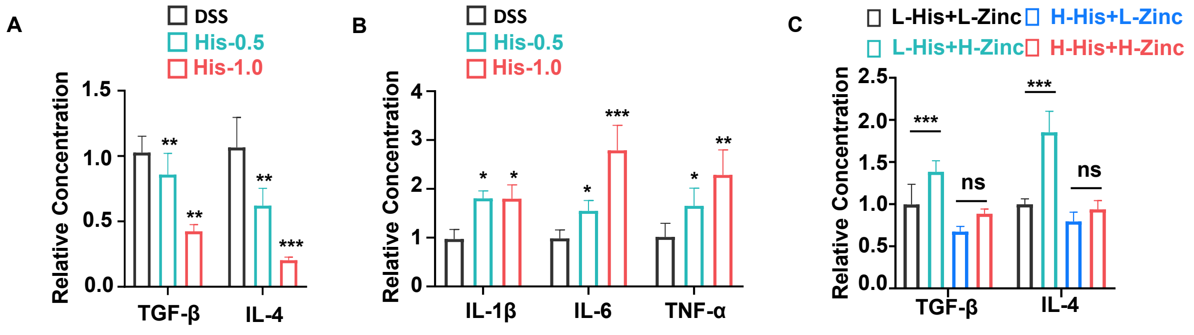

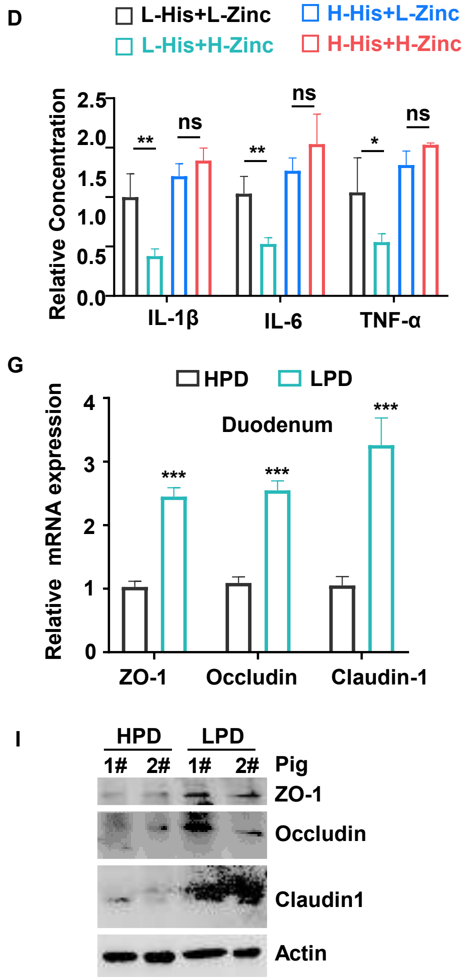

E

Nutrients	HPD	LPD
CP(%)	20.74	17.72
NE(kcal/kg)	2448	2448
Ca(%)	0.80	0.80
AP(%)	0.35	0.35
SID Lys(%)	1.35	1.35
SID Met(%)	0.47	0.52
	0.73	0.73
SID Thr(%)	0.84	0.84
SID Trp(%)	0.29	0.28
SID Val(%)	0.85	0.85
SID Ile(%)	0.70	0.63
SID Arg(%)	1.27	1.00
SID His(%)	0.46	0.38
SID Phe(%)	0.85	0.68
SID Tyr(%)	0.60	0.48
SID Phe+Tyr(%)	1.45	1.16

CP: Crude protein; NE: Net energy; Ca: Total calcium; AP: ATTD phosphorus; SID: Standardized Ileal Digestible; Lys: Lysine; Met: Methionine; SAA: Sulfur-containing amino acid; Thr: Threonine; Trp: Tryptophan; Val: Valine; Ile: Isoleucine; Arg: Arginine; His: Histidine; Phe: Phenylalanine; Tyr: Tyrosine

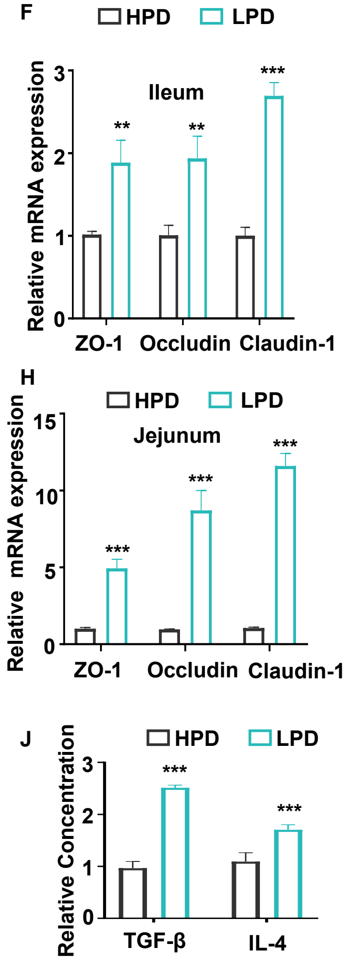

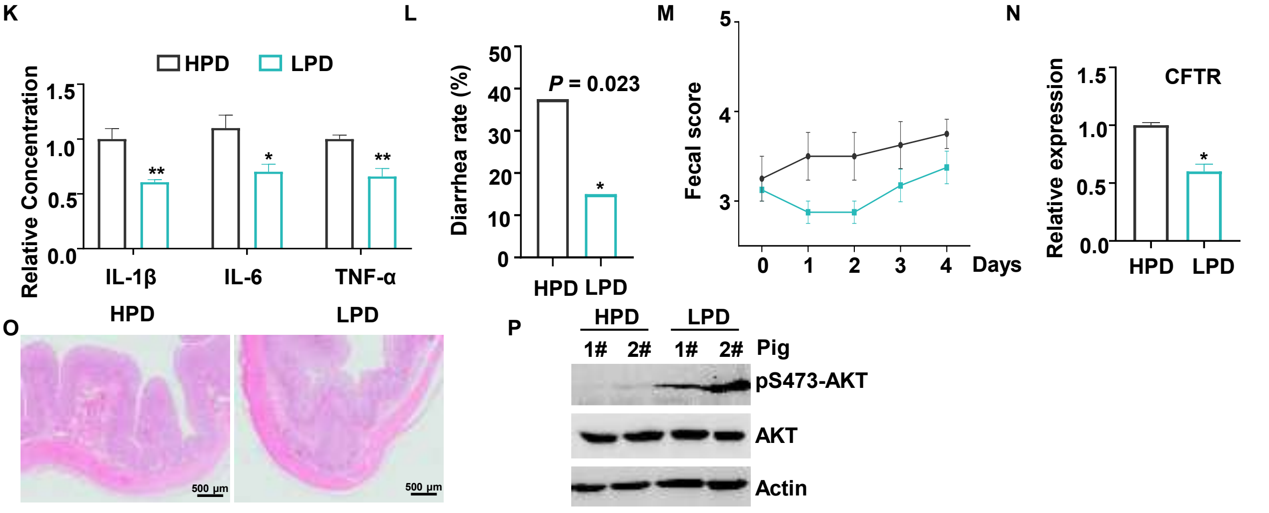

Fig. S5 Impact of histidine on zinc efficacy in reducing gut inflammation and barrier dysfunction.

(A). The expression levels of IL-4 and TGF-β in the colon tissue of mice were detected by ELISA. **(B).** The expression levels of IL-1β, IL-6 and TNF-α in the colon tissue of mice were detected by ELISA. **(C.** The expression levels of IL-4 and TGF-β in the colon tissue of mice were detected by ELISA. **(D).** The expression levels of IL-1β, IL-6 and TNF-α in the colon tissue of mice were detected by ELISA. **(E).** Comparison of diet composition analysis. **(F-H).** The expression levels of ZO-1, occludin and claudin-1 in the ileum tissue (F), duodenum (G) and jejunum tissue (H) of piglets from the HPD group and LPD treatment group were detected by qRT-PCR. **(I).** The expression levels of ZO-1, occludin and claudin-1 in the colon tissue of piglets from the HPD group and LPD treatment group were detected by WB. **(J).** The mRNA expression levels of IL-4 and TGF-β in the colon tissue of piglets from the HPD group and LPD group. **(K).** The expression levels of IL-1β, IL-6 and TNF-α in the colon tissue of piglets from the HPD group and LPD group were detected by ELISA. **(L).** Statistics of piglet diarrhea rate from the HPD group and LPD group. **(M).** Statistics of piglet fecal score from the HPD group and LPD treatment group. **(N).** The expression levels of CFTR in the colon tissue of piglets from the HPD group and LPD group were detected by qRT-PCR. **(O).** H&E staining of colon tissue in piglets from the HPD group and LPD group. **(P).** AKT activity in piglet colon tissue was detected by WB. （*$P<0.05$, **$P<0.01$, ***$P<0.001$）

Table S1 Primer sequences for qRT-PCR

species	Gene	Forward primer sequence (5'→3')	Reverse primer sequence (5'→3')
Mouse	*Zo-1*	ATTCAGGTCGCTCGCATGAC	ATTCAGGTCGCTCGCATGAC
	MUC2	ACATCTCCGTGTCTTACCTGG	GGGTCATGTTAAGAATCTTCCCTTC
	Claudin-1	TGTTTGCAGAGACCCCATCA	ACTAGAAGGTGTTGGCTTGGG
	Occludin	GCTCCTCAGCCAGCGTACTR	ATAGCTCTGTCCCAAGCAA
	IL-6	AATAGTCCTTCCTACCCCAA	GCTTAGGCATAACGCACT
	TNFa	AGGCACTCCCCCAAAAGATG	AACTGCACTACAGGCTCCGAGA
	IL-1β	AACTGCACTACAGGCTCCGAGA	GCCACAGGTATTTTGTCGTTGCTT
	IL-4	ATCATCGGCATTTTGAACGAGGTC	ACCTTGGAAGCCCTACAGACGA
	IL-10	GCCGGGAAGACAATAACTGC	GCCTGGGGCATCACTTCTAC
	TGFβ	TGCGCTTGCAGAGATTAAAA	CTGCCGTACAACTCCAGTGA
	β-actin	TACGCCAACACGGTGCTGTC	GTACTCCTGCTTGCTGATCCACAT
Human	*ZO-1*	AGCCCGAGGCGTGTTT	GGTGGGAGGATGCTGTTG
	Occludin	GCACCCAGCAACGACAT	CATAGACAGAATCCGAATCAC
	Claudin-1	AAGGACAAAACCGTGTGGGA	CTCTCCCCACATTCGAGATGATT
	CFTR	ACTATGGACCCTTCGAGCCT	CGCATTTGGAACCAGCGTAG
	ZNG1	GGCCTTTCCCGTATTGCTCA	TTCTCCAGCGCACTTCCTTC
	METAP1	CATCCAGGGCTCGTACTTCTG	TCTCGCTTCGCCTTTTCATCT
	β-Actin	TGCGGGACATCAAGGAGAAGC	ACAGCACCGTGTTGGCGTAGAG
	PRMT5	AGAGGATTGCAGTGGCTCTTG	TGGTTGGTGCCTGTGATGAT
Pig	*Claudin-1*	TTACTCCTACGCTGGTGACAACATTG	TGGATCTGCCCGGTGCTCTG
	Occludin	CAGTGGTAACTTGGAGGCGTCTTCR	GTGTAGTCTGTCTCGTAATGGTCTTC
	ZO-1	TCCTGAGTTTGATAGTGGCGTTGAC	CACGGTGTGACCATCCTCATCTTC
	MUC2	ACAGCTGCTCCTGCTGCAAA	CTGGCAGCTCTCGATGTGGG
	CFTR	ACTATGGACCCTTCGAGCCT	CGCATTTGGAACCAGCGTAG
	ZNG1	ACAGTCTCTCTGGAATAAGTTTGC	ACAGTCTCTCTGGAATAAGTTTGC
	TLR2	TCATCTCCCAAATCTGCGAAT	GGCTGATGTTCTGAATTGACCTC
	TLR4	CCGTCATTAGTGCGTCAGTTCT	CCGTCATTAGTGCGTCAGTTCT
	IL-6	CCATTCGGATAATGTAGCTG	ACATAAAATATTTCAAGTGGC
	IL-8	TTCGATGCCAGTGCATAAATA	CTGTACAACCTTCTGCACCCA
	IL-12	CGTGCCTCGGGCAATTATA	CGCAGGTGAGGTCGCTAGTT
	IL-4	GCTGCCCCAGAGAACACGAC	AGGTTCCTGTCAAGTCCGCTC
	TGFβ	TCCAAGGACCCTTCTCGGAT	AAAAACCGAGATGGGCGAGA
	METAP1	CAGTCTCTCTGGAATAAGTTTGC	AGCCCCTTTAGCCGTATGAC
	β-Actin	GATCTGGCACCACACCTTCTACAAC	TCATCTTCTCACGGTTGGCTTTGG
	PRMT5	GGACATCACTCTGAGTATCCGT	GACACAGATGGTCTGGCCTT
